# Plasma proteomic profiles of lung volume–based phenotypes in tobacco-exposed individuals without spirometric chronic obstructive pulmonary disease

**DOI:** 10.1093/annalsats/aaoag051

**Published:** 2026-03-03

**Authors:** Siyang Zeng, Claire Guo, Katherine A Pratte, Gang Luo, Russell P Bowler, Mehrdad Arjomandi

**Affiliations:** Department of Medicine, University of California, San Francisco, CA, United States; Department of Biomedical Informatics and Medical Education, University of Washington, Seattle, WA, United States; Department of Biostatistics & Informatics, University of Colorado, Anschutz Medical Campus, Aurora, CO, United States; Department of Genomic Sciences and Systems Biology, National Jewish Health Systems, Denver, CO, United States; Department of Biomedical Informatics and Medical Education, University of Washington, Seattle, WA, United States; Department of Systems Biology and Genome Sciences, Cleveland Clinic, Cleveland, OH, United States; Department of Medicine, University of California, San Francisco, CA, United States

## Abstract

**Background:**

Among individuals with a history of smoking but preserved spirometry (tobacco exposed with preserved spirometry, or TEPS), lung volume–based stratification identifies 2 phenotypes at increased risk for chronic obstructive pulmonary disease (pre-COPD): those with a relatively elevated total lung capacity ([TLC]^high^) and those with a relatively elevated functional residual capacity-to-TLC ratio ([FRC/TLC]^high^). These subgroups exhibit distinct respiratory symptoms, radiographic abnormalities, and clinical trajectories.

**Objective:**

This study aimed to determine whether these lung volume–based pre-COPD phenotypes have distinct biological features reflected in their circulating proteomes.

**Methods:**

We analyzed peripheral blood proteomic profiles (SomaScan v4.0; 4979 aptamers) from 1959 TEPS participants at the 5-year follow-up visit (visit 2) of the Genetic Epidemiology of COPD study cohort. Participants with [TLC]^high^ and [FRC/TLC]^high^ (based on computerized tomography scan-derived supine lung volumes) were compared with a low-COPD-risk reference group (without high TLC or high FRC/TLC). Analyses included covariate-adjusted regression, machine learning, and pathway enrichment modeling, with adjustment for age, sex, height, weight, smoking status and burden, leukocyte and platelet counts, forced expiratory volume in 1 second (percent predicted), and study site (random effect).

**Results:**

Using visit 2 data and visit 3 (10-year) follow-up outcomes, we confirmed the reproducibility and prognostic validity of the lung volume–based phenotypes in 1232 participants with longitudinal data. Over a mean (SD) of 5.3 (1.1) years, spirometric COPD developed in 17% (133 out of 761) of pre-COPD TEPS vs 8% (37 out of 471) of low-risk TEPS (adjusted odds ratio [aOR], 2.51; 95% confidence interval [CI], 1.69-3.75; *P* < .001). Among pre-COPD subgroups, [FRC/TLC]^high^ TEPS showed greater progression to a Global Initiative for Chronic Obstructive Lung Disease stage 2 or higher (aOR, 2.90; 95% CI, 1.62-5.18; *P* < .001) and preserved ratio and impaired spirometry (aOR, 3.29; 95% CI, 1.41-7.69; *P* = .005). At baseline (*n* = 1959), plasma proteomic analysis identified 165 upregulated and 145 downregulated proteins in [TLC]^high^ TEPS compared with low-COPD-risk TEPS, whereas only 22 proteins were differentially expressed in [FRC/TLC]^high^ TEPS vs low-risk group. Comparison between the 2 pre-COPD phenotypes identified 269 differentially expressed proteins (116 upregulated and 153 downregulated in [FRC/TLC]^high^ vs [TLC]^high^), including previously described COPD-related mediators (eg, soluble receptor for advanced glycation end products, insulin-like growth factor-binding protein) and novel candidates (eg, zymogen granule membrane protein 16). Pathway analysis highlighted immune signaling, cellular trafficking, and apoptotic pathways relevant to COPD pathogenesis.

**Conclusions:**

Lung volume–based stratification in TEPS identifies biologically distinct subgroups with differing plasma proteomic signature and COPD risk, underscoring the heterogeneity of early disease and revealing potential circulating biomarkers of pre-COPD states.

**Registration:**

COPDGene study is registered with ClinicalTrials.gov: ID NCT00608764.

Keywords

pre-COPD, lung volumes, proteomics, biomarkers, chronic obstructive pulmonary disease.

## Introduction

Chronic obstructive pulmonary disease (COPD) is a heterogeneous condition that develops in only a subset of individuals exposed to tobacco smoke.^1-4^ Among those who do, the rate of lung function decline varies widely.^5^^,^^6^ The factors driving this variable susceptibility are poorly understood, and identifying the “susceptible” individuals remains critical for prognostication, prevention, and early intervention.

Recent studies have shown that, among tobacco-exposed individuals with preserved spirometry (TEPS), lung volume indices can delineate distinct pre-COPD subgroups with varying susceptibility to disease development and progression.^7-11^ These subgroups not only display distinct baseline clinical and radiographic characteristics but also follow divergent disease trajectories.^7-11^ One subgroup is characterized by an elevated functional residual capacity to total lung capacity ratio (FRC/TLC) without increased TLC ([FRC/TLC]^high^), suggesting air trapping and airway disease in the absence of spirometric obstruction. The other is defined by high TLC values without an increased FRC/TLC ([TLC]^high^), indicating reduced lung elastance and thoracic expansion without airway disease, air trapping, or hyperinflation.^10^^,^^11^ Although these lung volume-based phenotypes have been described and reproduced across cohorts,^7-10^ skepticism about their validity persists, particularly given the limited understanding of their biological basis.

The objective of this study was to investigate the biological features associated with these lung volume–based phenotypes, as defined by FRC/TLC and TLC. We hypothesized that the differing susceptibility of TEPS with [FRC/TLC]^high^ vs [TLC^high^] to COPD development reflects distinct biological processes that can be detected through plasma proteomics. To test this hypothesis, we leveraged data from the Genetic Epidemiology of COPD (COPDGene) study in which we previously demonstrated the clinical validity of these phenotypes and examined peripheral blood proteomic signatures among TEPS.

## Methods

### Study design

**Figure 1:**
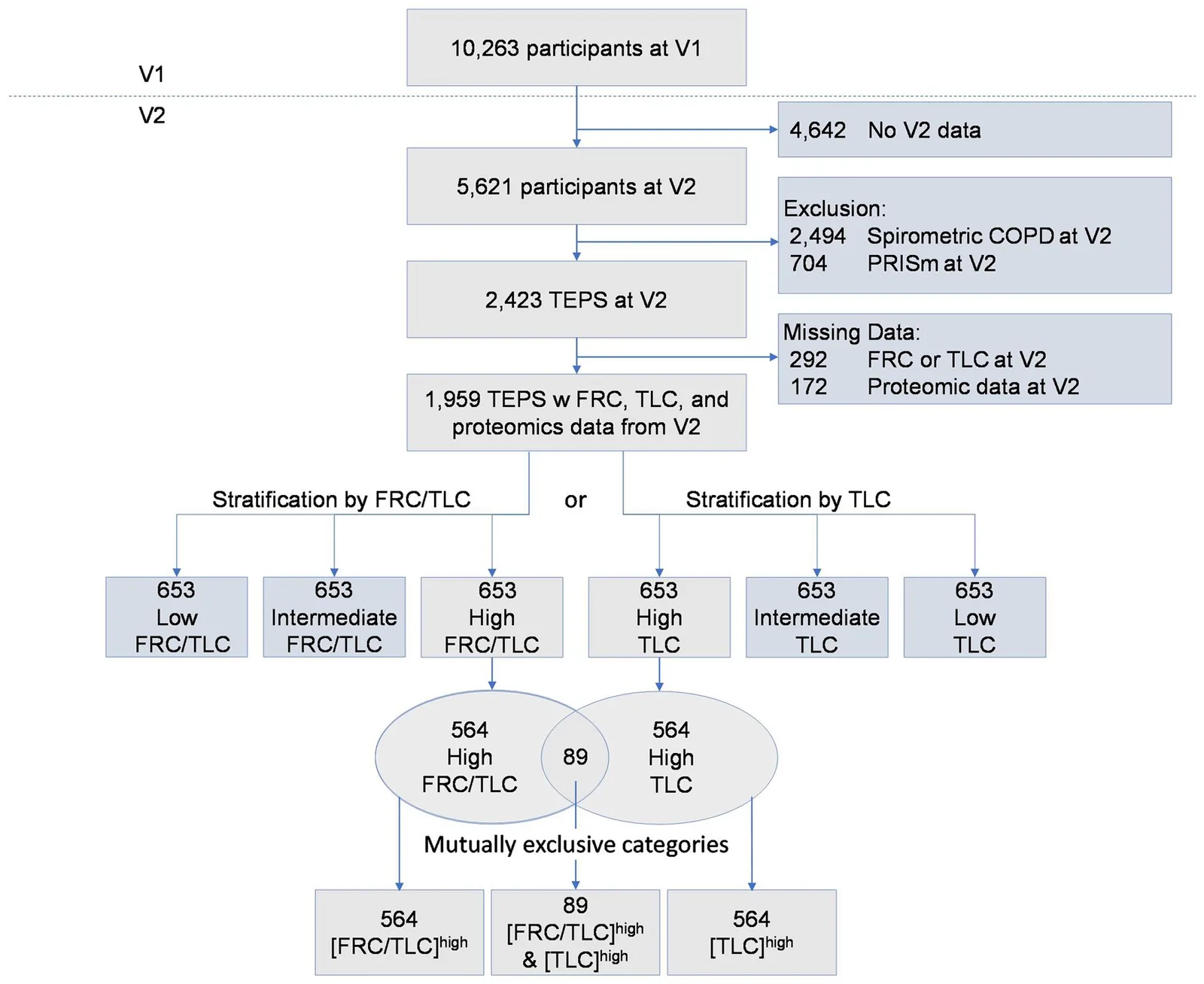
Participants flow through study protocol. Abbreviations: COPD, chronic obstructive pulmonary disease; FRC, functional residual capacity; [FRC/TLC]^high^, TEPS with high FRC/TLC but not high TLC; TEPS, persons with tobacco exposure and preserved spirometry; TLC, total lung capacity; [TLC]^high^, TEPS with high TLC but not high FRC/TLC; V1, baseline visit (visit 1); V2, visit 2.

COPDGene is a multicenter, prospective cohort study (2007-2010) of 10 263 current and former smokers aged 45-80 years, with or without COPD, excluding other active lung diseases except asthma, emphysema, and chronic bronchitis.^12^ Participants returned at 5 years (visit 2 [V2]) and 10 years (visit 3 [V3]). Plasma proteomics (SomaScan v4.0) were generated at V2. This analysis included V2 participants with computed tomography (CT)-derived supine lung volumes (TLC and FRC), pre- and postbronchodilator spirometry, and V2 proteomic data. TEPS was defined by postbronchodilator forced expiratory volume in 1 second (FEV_1_)/FVC of at least  0.70 and FEV_1_  of at least 80% predicted.^13^ TEPS with preserved ratio and impaired spirometry (PRISm) at V2 were excluded. V3 provided longitudinal outcomes (Figure 1).

Because validated reference equations for CT-derived supine position lung volumes are lacking, V2 CT-derived TLC and FRC were adjusted for age, sex, height, and weight before tertile stratification.^7^^,^^10^ Mutually exclusive categories were then created: (1) high TLC but not high FRC/TLC ([TLC]^high^), (2) high FRC/TLC but not high TLC ([FRC/TLC]^high^), and (3) both high TLC and high FRC/TLC ([FRC/TLC]^high^ and [TLC]^high^).^10^

### CT indices of lung volumes, air trapping, emphysema, and small airways

Computed tomography metrics included CT-measured TLC and FRC and their ratio, indices of air trapping (percent of lung voxels on expiratory CT with attenuation ≤ -856 Hounsfield units [HU]^14^^,^^15^ and parametric response mapping of air trapping [PRM^air trapping^]);^16^^,^^17^ indices of emphysema (percent of lung voxels on inspiratory CT with attenuation ≤ -950 HU and PRM of emphysema [PRM^EMPH^]);^16^^,^^17^ and measures of airway disease (mean value for the square root of wall area of a hypothetical airway with 10 mm internal perimeter [Pi10]).^18^

### SomaScan

Visit 2 plasma samples were analyzed using SomaScan v4.0, comprising 4979 Aptamers mapping to 4776 unique human proteins. Full details are available in Supplemental Methods.^19^

### Data management and statistical analysis

The workflow (**Figure S1**) integrated data preprocessing, mixed-effect modeling, machine learning, principal component analysis, and pathway enrichment analyses, performed in R (v4.2.2; R Foundation for Statistical Computing, Vienna, Austria) and visualized in GraphPad Prism (v9.0.0; GraphPad Software, San Diego, CA, USA). Covariates were selected based on conventional confounders relevant to each analysis. Lung volume measures were adjusted for age, sex, height, and weight.^9^ Outcome analyses were additionally adjusted for smoking status (current vs former), smoking burden (pack-years), and follow-up time.^20^ Longitudinal outcome analyses also included corresponding baseline measurements to account for preexisting differences. Proteomic analyses were adjusted for age, sex, height, weight, smoking status, and burden, as well as total leukocyte and platelet count, per SomaLogic recommendations for plasma proteomic SomaScan data based on prior internal quality control.^21^^,^^22^ Moreover, FEV_1_ percent predicted was included to account for within the normal range variations among TEPS.^7-11^

#### Lung volume stratification and longitudinal analyses

Regression models used lung volumes as continuous and tertile variables.^7^ Spirometric COPD at visit 3 was assessed using mixed-effect logistic models. Global Initiative for Chronic Obstructive Lung Disease (GOLD) stage distributions were compared using 1-way analysis of variance with Tukey–Kramer post hoc testing. Baseline and longitudinal outcomes including CT-derived indices, bronchodilator responsiveness, 6-Minute Walk Distance (6MWD), symptoms (modified Medical Research Council Dyspnea Scale [mMRC]), and Saint George’s Respiratory Questionnaire [SGRQ]), and self-reported severe exacerbations were examined using mixed-effect logistic or linear modeling with covariate adjustment.

#### Proteomics data pre-preprocessing and analyses

After integrating CT, lung function, and proteomic data, variance stabilization normalization^23^ was applied. Participants with missing values were excluded.

Three separate analyses were performed comparing (1) the 2 pre-COPD phenotypes of [TLC]^high^ and [FRC/TLC]^high^ (primary analysis); (2) [TLC]^high^ and low-COPD risk; and (3) [FRC/TLC]^high^ and low-COPD risk. Mixed-effect linear regression was used to identify differentially expressed proteins with multiple testing correction using the Benjamini–Hochberg false discovery rate (FDR) method with an FDR less than 0.05 being significant. Residuals from similar models (excluding the phenotype as a variable) were computed for each protein to obtain the adjusted protein expression levels.

Differentially expressed protein were *z*-normalized and visualized in heatmap. Unsupervised hierarchical clustering of the heatmap was used to examine the differences between the phenotypes based on dendrograms and adjusted protein expression. Differentially expressed proteins associated with the phenotype were examined by the sign and magnitude of the parameter estimate (adjusted fold change) using volcano plots.

#### Machine-learning analysis

To evaluate protein contributions to phenotype differentiation, machine-learning analysis of the significantly expressed proteins was performed, inclusively in one model, to predict the phenotypes. A model was obtained from comparing several candidate models constructed with 3-, 4-, or 5-fold nested cross-validation^24^ and 1 of the 3 widely used machine-learning algorithms (XGBoost, Random Forest, and Naive Bayes). Feature importance was estimated from each model to quantify each protein’s contribution (as a percentage) to outcome prediction.

#### Pathway enrichment analysis

Pathway enrichment analysis was performed via active subnetwork search in protein-protein interaction networks including Kyoto Encyclopedia of Genes and Genomes (KEGG) and Gene Ontology. Differentially expressed proteins were used to identify phenotype-associated interacting protein subnetworks along with enriched biological pathways containing these proteins. Pathways were hierarchically clustered using kappa statistics, and network plots were generated to visualize relationship among clustered pathways.

## Results

### Participant characteristics at V2

Because SomaScan v4.0 proteomics data were only available from blood samples collected at V2, we focused our analysis on the subset of the overall cohort who participated in V2, using V2 as the baseline visit and V3 as the follow-up visit (Figure 1). From the original 5621 V2 participants in COPDGene study, 2423 had smoking history and preserved spirometry (TEPS or GOLD stage 0). Of those, 1959 had baseline (V2) data and 1232 had follow-up (V3) data available. Table 1 shows the characteristics of those participants, who were 53% female, with a mean (SD) age of 64 (8) years; 38% were current smokers, with a mean (SD) smoking history of 38 (21) pack-years. Baseline characteristics of the 727 participants without follow-up data were comparable to those with follow-up data (**Table S1**). Categorization of TEPS by tertiles of TLC and FRC/TLC generated 4 groups: (1) [FRC/TLC]^high^ (*n* = 564); (2) [TLC]^high^ (*n* = 564); (3) [TLC]^high^ and [FRC/TLC]^high^ (*n* = 89), all high-COPD-risk; and (4) low-COPD-risk (*n* = 742) (Table 1).

**Table 1:** Characteristics of TEPS from Genetic Epidemiology of COPD visit 2 participants.

Characteristics	High-COPD-risk (pre-COPD)			Low-COPD-risk	All TEPS
	[FRC/TLC]^high^	[FRC/TLC]^high^ and [TLC]^high^	[TLC]^high^		
**Demographic**					
** V2 participants, No.**	564	89	564	742	1959
** Age, mean (SD), y**	62.8 (8.7)	63.3 (8.4)	63.0 (7.6)	64.4 (8.5)	63.5 (8.3)
** Female, No. (%)**	305 (54.1)	44 (49.4)	283 (50.2)	401 (54.0)	1033 (52.7)
** Height, mean (SD), cm**	168.8 (9.4)	169.1 (11.6)	179.8 (9.6)	168.4 (9.5)	169.0 (9.6)
** Body mass index, mean (SD), kg/m^-2^**	29.2 (6.3)	27.3 (5.5)	29.6 (5.9)	29.2 (5.6)	29.3 (5.9)
** Current smoker, No. (%)**	283 (50.2)	50 (56.2)	164 (29.1)	246 (33.2)	743 (37.9)
** Smoking history, mean (SD), pack-years**	38.6 (20.4)	44.6 (20.1)	36.7 (23.0)	36.5 (19.0)	37.5 (20.7)
**Spirometry, mean (SD)**					
** FEV_1_, L**	2.51 (0.61)	2.81 (0.72)	2.98 (0.67)	2.58 (0.60)	2.69 (0.66)
** FEV_1_, % predicted^a^**	96 (11)	100 (13)	102 (13)	96 (11)	98 (12)
** FVC, L**	3.22 (0.79)	3.70 (0.94)	3.87 (0.85)	3.27 (0.77)	3.45 (0.8)5
** FVC, % predicted^a^**	94 (12)	100 (13)	100 (12)	93 (11)	96 (12)
** FEV_1_/FVC**	0.78 (0.05)	0.76 (0.05)	0.77 (0.04)	0.79 (0.05)	0.78 (0.05)
** FEV_1_/FVC, % predicted^a^**	101 (6)	100 (6)	101 (6)	103 (7)	102 (6)
** FEF_25%-75%_,L**	2.40 (0.92)	2.35 (0.86)	2.65 (0.98)	2.57 (0.98)	2.53 (0.96)
**Bronchodilator responsiveness by FEV_1_, mean (SD), mL**	86.9 (169)	108 (138)	111 (151)	83.5 (149)	93.6 (156)
**Bronchodilator responsiveness by FEV_1_, mean (SD), %**	4.03 (7.36)	4.58 (6.24)	4.26 (6.11)	3.68 (6.53)	3.99 (6.66)
**Bronchodilator responsiveness by FEV_1_, No. (%)**	58 (10.3)	5 (5.6)	44 (7.8)	53 (7.1)	160 (8.2)
**CT indices, mean (SD)**					
** FRC, L**	2.86 (0.57)	3.82 (0.68)	2.95 (0.58)	2.42 (0.48)	2.77 (0.64)
** TLC, L**	4.57 (0.96)	6.27 (1.19)	6.37 (1.12)	5.01 (0.94)	5.33 (1.26)
** FRC/TLC, %**	63 (5)	61 (5)	47 (5)	49 (5)	53 (9)
** Inspiratory capacity, L**	1.71 (0.49)	2.45 (0.63)	3.42 (0.73)	2.59 (0.57)	2.57 (0.89)
** HU ≤ −950, %**	5.1 (8.0)	12.1(14.5)	14.3 (16.9)	8.51 (11.6)	9.35(13.2)
** PRM^EMPH^, %**	0.56 (1.2)	1.6 (2.3)	0.9 (1.6)	0.6 (1.4)	0.7 (1.5)
** HU ≤ −856, %**	66.0 (45.8)	109.4 (59.3)	54.9 (39.1)	40.0 (30.5)	54.9 (42.5)
** PRM^air trapping^, %**	11.3 (8.8)	18.0 (10.3)	8.9 (5.8)	7.4 (6.5)	9.4 (7.7)
** Pi10, %**	2.2 (0.4)	2.0 (0.4)	1.8 (0.3)	1.9 (0.3)	1.9 (0.4)
**Visit 3**					
** Participants, No.**	322	46	393	471	1232
** Follow-up, mean (SD), y**	5.4 (1.3)	4.9 (1.0)	5.3 (1.1)	5.3 (1.1)	5.3 (1.1)
** Progression to PRISm**					
** Progression to COPD, No. (%)**	56 (9.9)	14 (15.7)	63 (11.2)	37 (5.0)	170 (8.7)
** Annualized change in FEV_1_, mean (SD), mL**	−24.4 (25.4)	−21.0 (22.9)	−20.8 (22.4)	−18.6 (21.1)	−20.9 (22.9)
** Annualized % change in FEV_1_, mean (SD), %**	−1.26 (1.46)	−0.91 (0.94)	−0.83 (0.90)	−0.90 (1.06)	−0.97 (1.14)
** Annualized change in FVC, mean (SD), mL**	−21.9 (32.5)	−12.1 (27.8)	−17.0 (32.4)	−17.2 (29.0)	−18.2 (31.1)
** Annualized % change in FVC, mean (SD), %**	−0.86 (1.23)	−0.40 (0.83)	−0.56 (0.98)	−0.66 (1.07)	−0.67 (1.08)
**Annualized % change in FEV_1_/FVC, mean (SD), %**	−0.38 (0.82)	−0.51 (0.68)	−0.29 (0.65)	−0.25 (0.61)	−0.31 (0.69)

Abbreviations: COPD, chronic obstructive pulmonary disease; CT, computed tomography; FEF25-75, maximum airflow at mid-lung volume; FEV1, forced expiratory volume in 1 second; FVC, forced vital capacity; FRC, functional residual capacity; HU ≤ -856,  percentage of the lung voxels with attenuation ≤ −856 Hounsfield Units on the expiratory CT images; HU ≤ -950, percentage of the lung voxels with attenuation ≤ -950 Hounsfield Units on the inspiratory CT images; Pi10, the mean for the square root of wall area of a hypothetical airway with 10 mm internal perimeter; PRISm, preserved ratio and impaired spirometry; PRM^air trapping^, parametric response mapping of percent air trapping; PRM^EMPH^, parametric response mapping of functional small airway disease as measures of emphysema; TLC, total lung capacity.

a Reference equations: percent predicted of normal values of spirometry and lung volumes were calculated using Third National Health and Nutrition Examination predicted formulas, respectively. Bronchodilator responsiveness was defined as at least 12% and at least 200 mL increase in FEV_1_ after bronchodilators administration.

### Disease characteristics and progression in TEPS from V2 to V3

**Figure 2:**
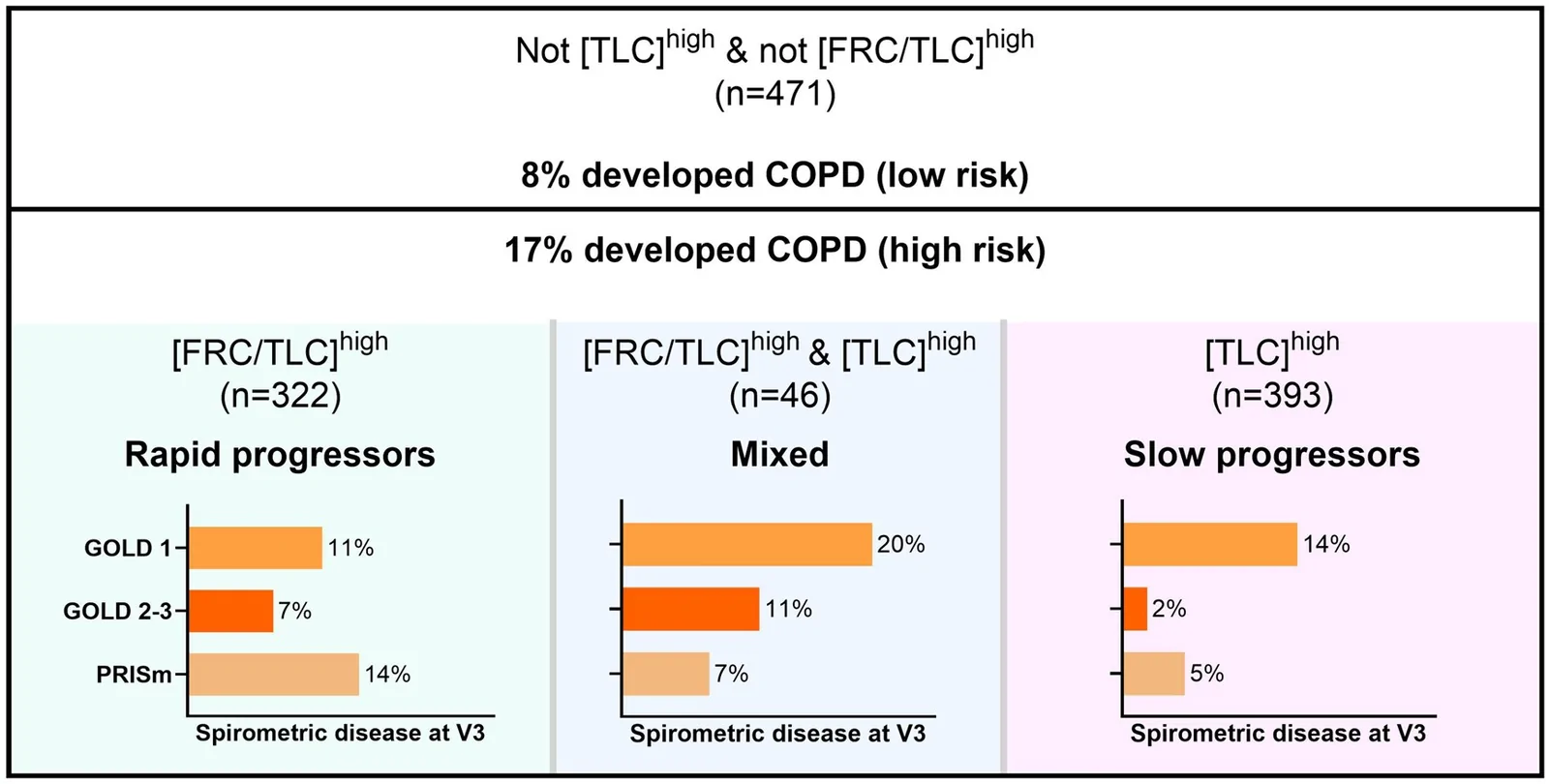
Spirometric progression of TEPS at 5-year follow-up visit stratified by lung volumes. Bar plots show the distribution of COPD GOLD stage at 5-year follow-up (V3) visit with lung volume–based phenotypes determined at V2. The lung volumes were adjusted for age, sex, height, and weight. GOLD stage 4 was omitted because no participants developed GOLD stage 4. Abbreviations: COPD, chronic obstructive pulmonary disease; FRC, functional residual capacity; GOLD, Global Initiative for Chronic Obstructive Lung Disease; TEPS, persons with tobacco exposure and preserved spirometry; TLC, total lung capacity; V2, visit 2; V3, visit 3.

**Figure 3:**
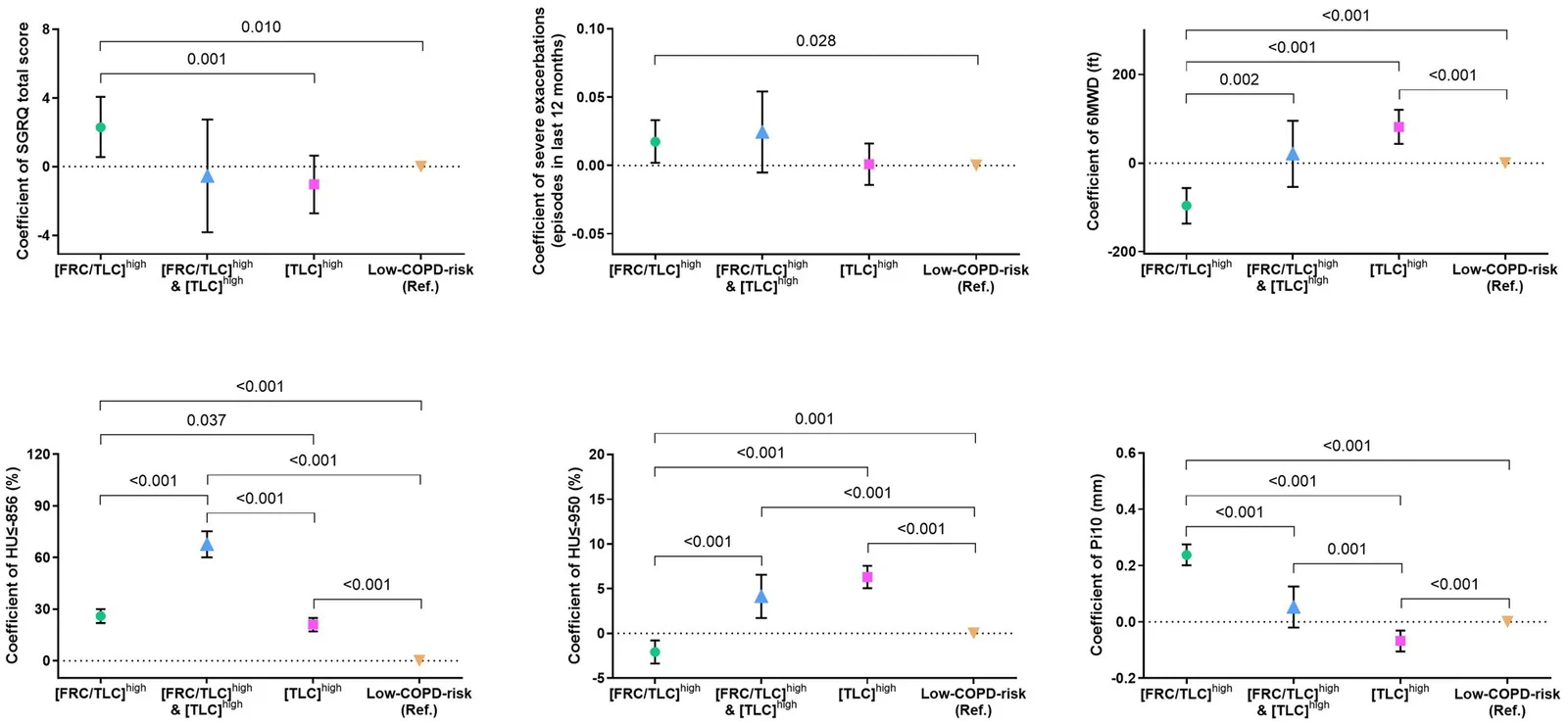
Baseline clinical characteristics and radiographic features among TEPS stratified by lung volume phenotypes. The points and the bars represent coefficients and 95% confidence intervals from adjusted regression models of clinical characteristics and radiographical features for the phenotypes [FRC/TLC]^high^, [FRC/TLC]^high^ and [TLC]^high^, [TLC]^high^, and low-COPD-risk (reference). Abbreviations: 6MWD, 6-Minute Walk Distance Test; COPD, chronic obstructive pulmonary disease; FRC, functional residual capacity; [FRC/TLC]^high^, TEPS with high FRC/TLC but not high TLC; Pi10, mean square root of wall area of a hypothetical airway with 10 mm internal perimeter; PRM^air trapping^, PRM for air trapping; PRM^EMPH^, parametric response mapping for emphysema; SGRQ, St George’s Respiratory Questionnaire; TEPS, persons with tobacco exposure and preserved spirometry; TLC, total lung capacity; [TLC]^high^, TEPS high TLC but not high FRC/TLC.

Over a mean (SD) of 5.3 (1.1) years from V2 to V3 (median [25th-75th percentile] = 5.1 [4.6-6.0] years), 8% of low-COPD-risk TEPS and 17% of high-COPD-risk TEPS developed spirometric COPD (adjusted odds ratio [aOR], 2.51; 95% confidence interval [CI], 1.69-3.75; *P* < .001). Within the high-risk stratum, [FRC/TLC]^high^ participants were more likely than [TLC]^high^ participants to experience faster spirometric progression to GOLD stages of at least 2 (aOR, 3.29; 95% CI, 1.41-7.69; *P* = .005) and to PRISm (aOR, 2.90; 95% CI, 1.62-5.18; *P* < .001) (Figure 2 and **Figure S2**). Furthermore, among high-COPD-risk TEPS, [FRC/TLC]^high^ participants had more respiratory symptoms (mMRC and SGRQ), reduced exercise tolerance (6MWD), greater air trapping (HU ≤ -856 and PRM^air trapping^), increased airway wall thickness (Pi10), and more bronchodilator responsiveness, whereas [TLC]^high^ participants had more emphysema (by HU ≤ -950 and PRM^EMPH^) with fewer symptoms and better 6MWD. Compared with low-risk TEPS, [FRC/TLC]^high^ showed higher odds of symptoms (SGRQ), severe exacerbations, bronchodilator responsiveness, air trapping (HU ≤ -856 and PRM^air trapping^), and thicker airways (Pi10). In contrast, [TLC]^high^ showed more air trapping and emphysema but similar symptoms and exacerbation and better 6MWD. These findings are summarized in Figure 3 with full metrics shown in **Figures S3 and S4**.

### Proteomics analysis between pre-COPD phenotypes

**Figure 4:**
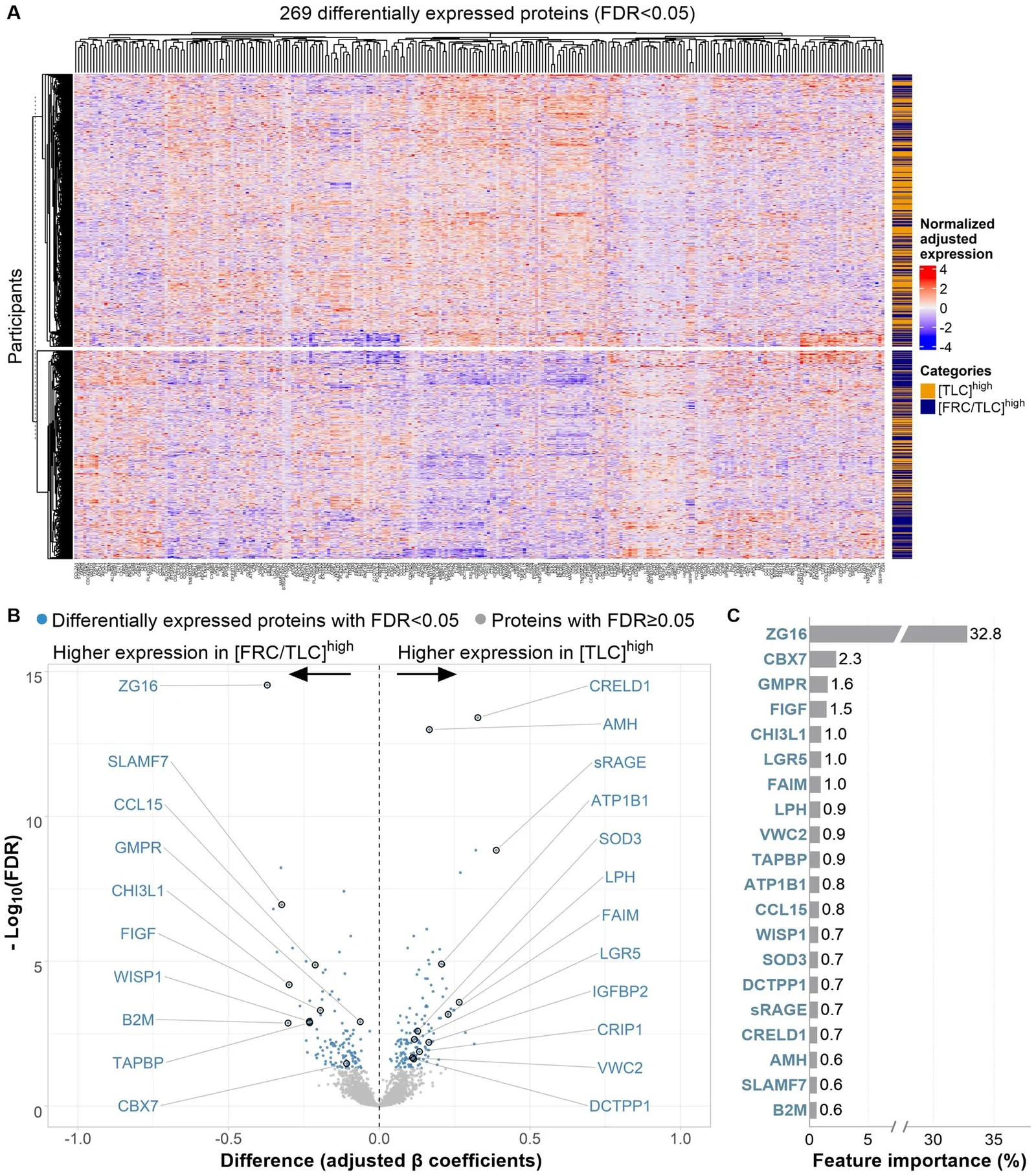
Phenotype association and machine-learning results for phenotypes [TLC]^high^ vs [FRC/TLC]^high^. The difference in plasma proteomics data between phenotypes [TLC]^high^ (*n* = 564) vs [FRC/TLC]^high^ (*n* = 564) was examined using mixed-effect linear regression modeling with adjustment for covariates (age, sex, height, weight, smoking status [current vs former], smoking burden [pack-years], FEV_1_% predicted, white blood cell count, platelet count, and random effect of study site). Differentially expressed proteins for the phenotypes were determined by a FDR less than 0.05. Machine-learning analysis was performed to evaluate and rank the differentially expressed proteins for their “feature importance” (ie, contribution as a percentage to the outcome prediction) in distinguishing the phenotypes. (A) Heatmap of unsupervised clustering of adjusted expressions of the differentially expressed proteins for [TLC]^high^ vs [FRC/TLC]^high^. The heatmap was horizontally divided into 2 sections by using 2-means clustering before hierarchical clustering on the rows and column. (B) Volcano plot for the FDR and β coefficients of all 4979 proteins compared between [TLC]^high^ and [FRC/TLC]^high^. Differentially expressed proteins were marked in blue. Differentially expressed proteins that were ranked top 20 by the machine-learning analysis are labeled with protein symbols. (C) Bar plots for the “feature importance” of the ranked top 20 differentially expressed proteins for distinguishing between the phenotypes [TLC]^high^ and [FRC/TLC]^high^. Abbreviations: FDR, false discovery rate; FEV_1_, forced expiratory volume in 1 second; FRC, functional residual capacity; [FRC/TLC]^high^, TEPS with high FRC/TLC but not high TLC; TEPS, persons with tobacco exposure and preserved spirometry; TLC, total lung capacity; [TLC]^high^, TEPS high TLC but not high FRC/TLC; TLC, total lung capacity.

To investigate systemic biological differences between phenotypes associated with slower versus more rapid disease progression among high-COPD-risk TEPS, we compared plasma proteomic profiles between [TLC]^high^ and [FRC/TLC]^high^ participants. This analysis identified 269 differentially expressed proteins (FDR < 0.05) with 116 upregulated and 153 downregulated in [FRC/TLC]^high^ (Figure 4A and 4B). Supervised machine learning ranked the relative “feature importance” of these proteins in distinguishing the 2 phenotypes (Figure 4C), yielding an area under the receiver operating characteristic curve (ROC AUC) of 0.83 (95% CI, 0.80-0.85). To further assess the contribution of these proteins, we compared a covariate-only ROC model (age, sex, height, weight, smoking status, smoking burden, total leukocyte count, platelet count, and FEV_1_ percent predicted) with a model including both covariates and proteins. The addition of proteomic markers significantly improved model performance, increasing the AUC from 0.73 (95% CI, 0.70-0.76) to 0.87 (95% CI, 0.85-0.89) (**Table S2**).

Several proteins with established associations with COPD (including soluble receptor for advanced glycation end products [sRAGE], superoxide dismutase [SOD3], insulin-like growth factor-binding protein [IGFBP2], chitinase-3–like protein [CHI3L1], C–C motif chemokine ligand 1 [CCL15], WNT1-inducible signaling pathway protein 1 [WISP1], and c-Fos–induced growth factor [FIGF]) as well as novel candidates (zymogen granule protein 16 [ZG16], cysteine-rich with EGF-like domains 1 [CRELD1], lactase-phlorizin hydrolas [LPH], and von Willebrand factor C domain containing 2 [VWC2]) were among the top discriminators between phenotypes. Among proteins with known relevance to COPD, higher relative levels of sRAGE (an anti-inflammatory decoy receptor), SOD3 (an extracellular antioxidant enzyme), and IGFBP2 (a regulator of IGF signaling) were observed in [TLC]^high^ TEPS, whereas CHI3L1 (a mediator of inflammatory responses), CCL15 (a mononuclear cell chemoattractant), and WISP1 (a WNT pathway-associated mediator of airway remodeling) had higher expression in [FRC/TLC]^high^ TEPS. Notably, the novel protein zymogen granule protein 16 (ZG16) demonstrated the strongest discriminatory contribution, accounting for 32.8% of total feature importance and exceeding all other differentially expressed proteins by several folds (Figure 4C). Sensitivity analyses excluding participants who developed PRISm by V3 yielded consistent results, identifying 196 differentially expressed proteins of which 154 overlapped with the primary analysis (**Figure S5**).

**Figure 5:**
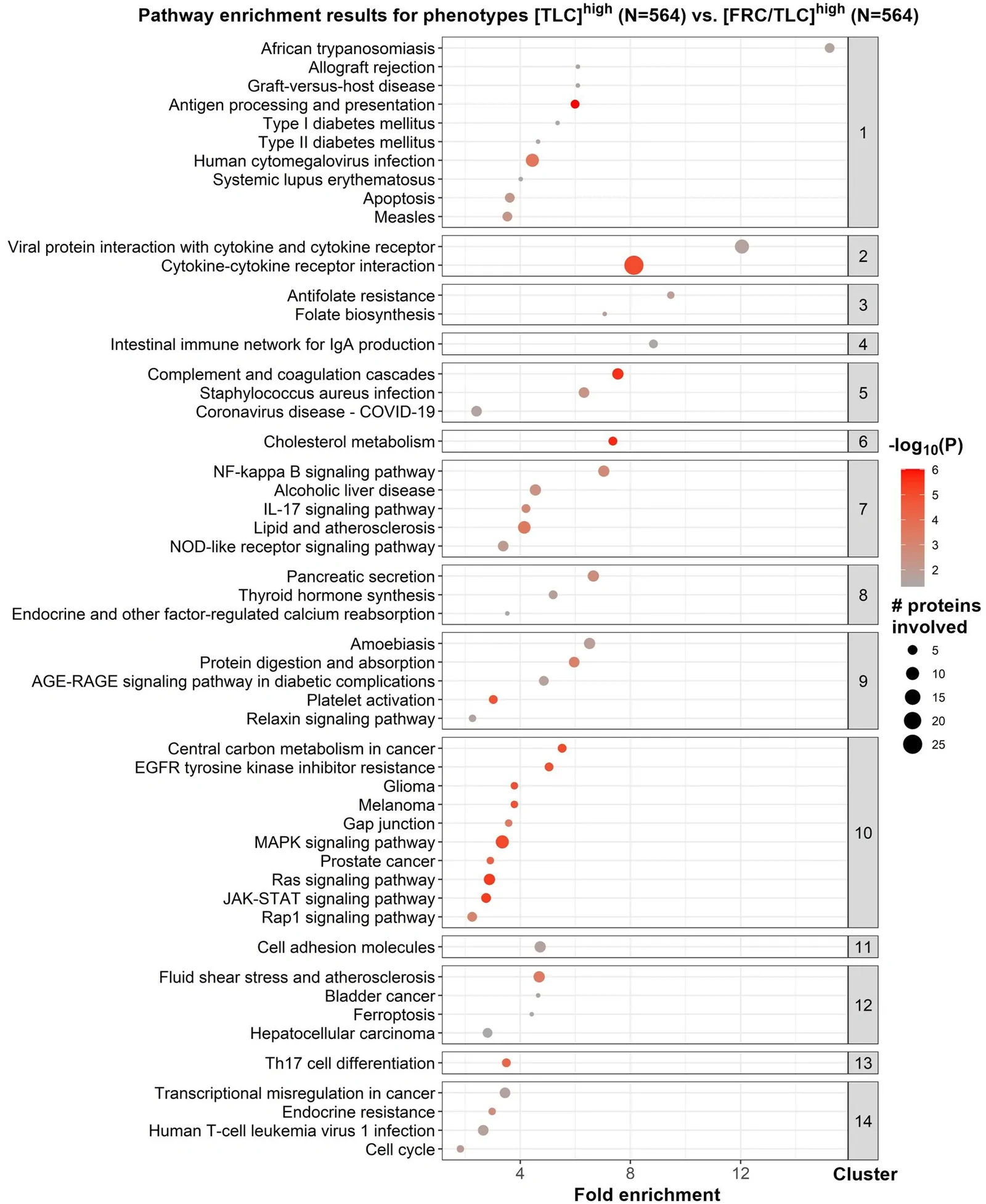
Bubble chart of pathway enrichment results for phenotypes [TLC]^high^ vs [FRC/TLC]^high^. Hierarchical clustering was applied to the enriched pathways for phenotypes [TLC]^high^ (*n* = 564) vs [FRC/TLC]^high^ (*n* = 564) based on kappa statistics about the differentially expressed proteins involved in each pathway.^25^ The x-axis corresponds to fold enrichment values, the rate of percentage of differentially expressed proteins belonging to the pathway over the percentage of the pathway’s proteins in the background.^25^ The y-axis lists the name of the top 10 pathways with the highest fold enrichment values in each cluster. Each panel with a numbered band on the right side denotes a cluster of pathways. The size of the bubble indicates the number of differentially expressed proteins involved in the given pathway. Color indicates the Bonferroni-adjusted *P* value of the enrichment by hypergeometric tests; the more it shifts to red, the more significantly the pathway is enriched. Abbreviations: FDR, false discovery rate; TEPS, persons with tobacco exposure and preserved spirometry; [FRC/TLC]^high^, TEPS with high FRC/TLC but not high TLC; TLC, total lung capacity; [TLC]^high^, TEPS with high TLC but not high FRC/TLC.

Pathway enrichment analysis implicated differential expression of multiple pro-inflammatory processes including immune cell signaling, trafficking, and activation, as well as secretory, hyperplastic, and apoptotic pathways with potential relevance to COPD pathogenesis (Figure 5 and **Figure S6**). To further delineate these differences, we examined the functional organization and directionality of the differentially expressed proteins. The [TLC]^high^ subgroup demonstrated a broader and more coordinated proteomic response, with upregulation of proteins linked to anti-inflammatory, epithelial-repair, and extracellular matrix homeostasis functions, including sRAGE, IGFBP2, and CRELD1. In contrast, the [FRC/TLC]^high^ subgroup exhibited a smaller number of changes dominated by proteins involved in complement activation, coagulation cascades, interleukin-17 signaling, nuclear factor (NF-κB) signaling, and NOD-like receptor pathways, canonical innate immune and host-defense cascades typically associated with inflammatory and epithelial injury responses. Correspondingly, pathway enrichment analysis of the [FRC/TLC]^high^ proteome highlighted complement and coagulation cascades, cytokine-cytokine receptor interaction, and viral response modules, indicating preferential activation of localized inflammatory programs. These results delineate 2 distinct systemic proteomic patterns: one broadly enriched for adaptive and tissue repair–associated proteins ([TLC]^high^) and another more narrowly dominated by innate inflammatory and immune activation pathways ([FRC/TLC]^high^).

### Proteomics of low-COPD-risk TEPS vs [TLC]^high^ and [FRC/TLC]^high^

Plasma proteomic comparisons between low-COPD-risk TEPS and the 2 pre-COPD phenotypes showed 252 differentially expressed proteins between low-COPD-risk and [TLC]^high^ (146 upregulated and 106 downregulated in [TLC]^high^) (**Figure S7**) but only 22 proteins between low-COPD-risk and [FRC/TLC]^high^ (10 upregulated and 12 downregulated in [FRC/TLC]^high^) (**Figure S8**).

Supervised machine-learning analyses identified distinct discriminatory protein panels for each comparison. For the low-risk vs [TLC]^high^ comparison, top-ranked proteins included sRAGE, IGFB2, and SPP1 (an immune modulator)—all high in [TLC]^high^—and ZG16, CCL8 (a monocyte chemoattractant), and CHI3L1 (involved in extracellular matrix remodeling), which were higher in low-risk participants, achieving an AUC of 0.73 (95% CI, 0.70-0.76) (**Figure S7**). For the low-risk vs [FRC/TLC]^high^ comparison, top-ranked proteins included TFRC (iron homeostasis and oxidative stress regulator), SERPINA1 (α_1_-antitrypsin), LRIG1 (epithelial regeneration and EGFR signaling), and C9 (complement activation component)—all in [FRC/TLC]^high^—while APOC3 (systemic inflammation regulator) and CRIP1 (immune cell modulator) were higher in low-risk participants, yielding an AUC of 0.63 (95% CI, 0.60-0.66) (**Figure S8**). Adding proteomic markers to a covariate-only model significantly improved discrimination between [TLC]^high^ and low-risk groups, increasing the AUC from 0.65 (95% CI, 0.62-0.68) to 0.76 (95% CI, 0.73-0.78). In contrast, inclusion of proteomic markers did not significantly enhance model performance for the [FRC/TLC]^high^ comparison (**Table S2**).

Pathway enrichment analysis showed that compared with low-risk TEPS, [TLC]^high^ subgroup displayed a broad proteomic activation pattern, encompassing multiple biological domains. Prominent pathways included metabolic and energetic processes (fatty-acid degradation, glycolysis/gluconeogenesis, carbon metabolism, HIF-1 signaling), inflammatory signaling networks (cytokine-cytokine receptor interaction, TNF, JAK-STAT, NOD-like receptor signaling, as well as antigen presentation; Th1/Th2/Th17 differentiation), and hemostasis and vascular mechanisms (complement and coagulation cascades, fluid shear stress, and atherosclerosis). Additional enrichment was observed in matrix and growth-factor networks (extracellular matrix receptor interaction, proteoglycans, cell adhesion/focal adhesion, MAPK, Ras, Rap1, and Hippo signaling). Finally, stress and protein turnover pathways (apoptosis, lysosome, ubiquitin-proteasome) and the AGE-RAGE axis were also upregulated, reflecting the broad systemic and tissue remodeling processes characterizing the [TLC]^high^ phenotype (**Figures S9 and S10**).

In contrast, compared with low-risk TEPS, the [FRC/TLC]^high^ subgroup demonstrated a more limited set of enriched pathways, comprising only 16 processes across 4 clusters, dominated by complement and coagulation cascades, with smaller signals in HIF-1, TGF-β, and ferroptosis pathways (FDR < 0.05). Using a more permissive threshold (FDR < 0.20) modestly expanded the enrichment profile to include 20 biological processes across 6 clusters, which remained predominantly hemostasis-related, with additional representation from systemic lupus erythematosus, several metabolic and secretory pathways, and a few developmental signaling categories (including mTOR, Hippo, WNT) (**Figures S11, S12, and S13**).

## Discussion

In prior work,^10^ we demonstrated that lung volume–based phenotyping of COPDGene participants with a history of smoking and preserved spirometry identified distinct spirometric, symptomatic, and radiographic disease patterns. In the present study, we extended this framework by leveraging plasma proteomic data from COPDGene V2 to determine whether these phenotypes exhibit distinct circulatory proteomic profiles. Because SomaScan 5K proteomic data were available only at V2, we first reproduced the previously established lung volume–based phenotypes to confirm the robustness of this classification. As expected, categorization by TLC and FRC/TLC again identified TEPS subgroups with low and high risks for developing spirometric COPD and, within the high-risk group, further differentiated slow and rapid progressors ([TLC]^high^ and [FRC/TLC]^high^, respectively). Using this validated framework, we identified plasma protein signatures associated with lung volume–based phenotypes, revealing known and novel immunomodulatory proteins. Consistent with these findings, when proteomic variables were added to covariate-only models distinguishing slow from rapid progressors within the high-risk pre-COPD group, model discrimination modestly improved (AUC, 0.73 to 0.87), underscoring the contribution of systemic proteomic signatures to the biological differentiation of these subtypes. Pathway enrichment analyses of these differentially expressed proteins revealed networks related to innate and adaptive immune signaling, leukocyte recruitment and activation, and epithelial secretory and remodeling processes, together with pathways regulating cellular proliferation and apoptosis, as well as inflammatory mechanisms and tissue injury–repair dynamics central to COPD pathogenesis. The distinct proteomic signatures and biological pathways observed across lung volume–based phenotypes, together with their divergent clinical and radiographic features, underscore the utility of lung volume measurements in identifying early COPD subtypes.

Although [FRC/TLC]^high^ and low-COPD-risk TEPS showed the greatest clinical and radiographic differences, only a few dozen proteins were differentially expressed between them, in contrast to the hundreds identified when comparing [TLC]^high^ TEPS with either groups. Because the [FRC/TLC]^high^ group exhibited more substantial airway abnormalities and symptom burden, we expected to observe greater divergence in their plasma proteomic profiles compared with the low-risk group. Surprisingly, the opposite pattern emerged, a finding unlikely to be attributable to random variation given the similar sample sizes across groups. One potential explanation is that the plasma proteomic profile observed in the [TLC]^high^ phenotype reflects activation of a more balanced or adaptive injury-repair response to smoking-related stress. Upregulated proteins in this group, including sRAGE (known to decline in COPD and functions as an anti-inflammatory decoy receptor)^26^ and IGFBP2 (shown in lung models to mediate alveolar-epithelial senescence and repair),^27^ are consistent with a systemic milieu favoring controlled repair rather than unchecked inflammation. These findings suggest that interindividual variability in systemic repair or adaptive capacity may contribute to divergent clinical trajectories among TEPS, wherein [TLC]^high^ individuals exhibit more emphysema yet fewer symptoms, while [FRC/TLC]^high^ individuals demonstrate greater airway disease burden but a muted systemic proteomic response. Alternatively, this pattern may reflect a more localized, airway-restricted inflammatory process, resulting in a blunted systemic signal in the [FRC/TLC]^high^ phenotype. Taken together, these observations suggest that slow-progressor ([TLC]^high^) individuals mount a coordinated systemic repair response that mitigates airway inflammation and symptom expression, whereas rapid-progressor ([FRC/TLC]^high^) individuals exhibit a more localized, airway-centered inflammatory process with limited systemic involvement.

Several of the differentially expressed plasma proteins warrant further examination. Notably, multiple proteins with known anti-inflammatory or antioxidant functions were elevated in [TLC]^high^ TEPS (slow progressors). Among them, sRAGE, a soluble receptor with anti-inflammatory and antioxidant properties, binds circulating AGEs, thereby preventing their interaction with membrane-bound RAGE.^28-30^ Prior studies have shown that higher plasma sRAGE levels are associated with protection against lung function decline and COPD progression,^31^ findings consistent with the clinical characteristics and trajectories of [TLC]^high^ phenotype. Another protein highly expressed in this group was SOD3, an extracellular antioxidant enzyme that mitigates oxidative stress and tissue injury, contributing to an anti-inflammatory milieu. IGFBP2, a regulator of insulin-like growth factor (IGF) signaling with key roles in cellular senescence and aging, was also elevated in [TLC]^high^ TEPS.^32-36^ IGFBP2 deficiency in mice has been linked to alveolar type 2 cell senescence and tissue repair,^27^ and other studies have implicated IGF-IGFBP2 signaling in the development of emphysema through effects on alveolar epithelial repair following smoking-induced injury.^37^ Given that COPD represents a disease of accelerated lung aging,^38^ higher IGFBP2 levels in [TLC]^high^ TEPS may reflect activation of accelerated repair mechanisms that counteract premature aging and preserve lung function. Collectively, these proteins and their associated biological pathways may help explain the slower rate of lung function decline observed in [TLC]^high^ TEPS.

In contrast, several proteins with proinflammatory and remodeling-associated functions were elevated in [FRC/TLC]^high^ TEPS (rapid progressors). These included CHI3L1 (YKL-40), a glycoprotein that mediates macrophage and epithelial inflammatory responses; CCL15, a chemokine that recruits mononuclear cells and promotes leukocyte trafficking to inflamed tissue; and WISP1, a WNT pathway-associated matricellular protein implicated in epithelial-mesenchymal transition, airway remodeling, and fibrotic repair. Together, these proteins suggest activation of innate immune and remodeling pathways that may underlie the airway-predominant pattern observed in rapid progressors. Another protein of interest, SERPINA1, exhibited higher expression in [FRC/TLC]^high^ TEPS compared with low-COPD-risk TEPS, although this difference did not reach statistical significance in comparison with [TLC]^high^ TEPS. SERPINA1 (alpha-1 antitrypsin) is a serine protease inhibitor that protects the lung from neutrophil elastase-mediated injury,^39^^,^^40^ and its deficiency is a well-established risk factor for early onset emphysema, particularly in smokers. ^41-43^ The relatively higher SERPINA1 expression observed in [FRC/TLC]^high^ TEPS may reflect a compensatory response to airway inflammation and protease activity, consistent with the lower degree of emphysema burden in this group.

A novel observation in our study was the identification of ZG16 as the most discriminative protein in the machine-learning models. ZG16 expression was higher in [FRC/TLC]^high^ TEPS compared with [TLC]^high^ TEPS and similarly elevated in low-COPD-risk TEPS compared with [TLC]^high^ TEPS, displaying a reverse pattern to that observed for sRAGE. ZG16, a member of the jacalin-related lectin family, binds mannose-rich glycans and is implicated in cellular recognition and immune regulation.^44^^,^^45^ While extensively characterized in the gastrointestinal tract, where it contributes to mucus barrier integrity by preventing bacterial adherence to the epithelial cell surface,^46^ ZG16 has not been studied in the context of pulmonary diseases. In animal models, ZG16 deficiency results in a denser, less permeable intestinal mucus layer,^47^ and emerging evidence suggests that ZG16 may influence dendritic cells maturation and immune activation.^44^ Given these immunomodulatory properties and its potential role in mucus regulation, elevated ZG16 expression in [FRC/TLC]^high^ TEPS may contribute to mucus hypersecretion and impaired clearance in chronic airway diseases, warranting further investigation.

This study has several limitations that should be considered. First, the unsupervised cluster analysis of proteomic data via heatmaps suggests that while lung volume–based stratification identifies TEPS at differential COPD risk, significant heterogeneity remains. The AUC of our machine-learning models, ranging from 0.61 to 0.82, suggests room for improvement. Thus, lung volume–based phenotyping may not fully capture COPD risk stratification in TEPS. A multidimensional approach incorporating lung function indices (TLC, FRC/TLC) alongside systemic biomarkers (eg, ZG16, sRAGE, IGFBP2) may provide a more precise framework for identifying pre-COPD and COPD phenotypes. Second, our data were derived from a single large research cohort (COPDGene), and while robust, our findings require replication in independent cohorts. However, most existing COPD cohorts lack the radiographic or physiologic data necessary to perform FRC- and TLC-based phenotyping. Recently, in a study of Subpopulations and Intermediate Outcome Measures in COPD Study (SPIROMICS) cohort, we demonstrated that lung volume–based phenotyping using residual volume (RV) and TLC similarly identifies distinct pre-COPD phenotypes.^11^ However, because SPIROMICS does not include FRC measurements and COPDGene does not include RV, direct comparison between cohorts is limited. In addition, ­although proteomic data (SomaScan 7K platform) are available in SPIROMICS, differences between the 7K and 5K platforms may limit direct replication. Future studies that harmonize lung volume phenotyping and proteomic approaches across cohorts will be critical to validate and extend our findings. Third, it is important to note that our study was designed to identify biological distinctions between lung volume–based phenotypes rather than to develop predictive models for future COPD. The proteins analyzed here were selected for cross-sectional phenotype discrimination, not for prediction of disease progression. Accordingly, these findings could provide context for potential biological pathways and systemic processes associated with heterogeneity among tobacco-exposed individuals without spirometric COPD, but they are not intended as a prognostic model. Notably, other proteomics studies of COPDGene cohort have focused on biomarker-based prediction of incident COPD and emphysema.^21^^,^  ^48^^,^^49^

## Conclusion

We demonstrate the utility of lung volume–based phenotyping (FRC/TLC and TLC) for identifying TEPS subgroups at risk for COPD, confirming our prior findings from COPDGene baseline visit data.^10^ These results, along with other studies,^7-9^^,^^11^ reinforce the concept of lung volume–based phenotyping as a framework for distinguishing pre-COPD subtypes with divergent symptoms, radiographic features, and clinical trajectories. Extending this framework, we show for the first time that these phenotypes also differ in their systemic proteomics signature, characterized by an adaptive, repair-enriched profile in [TLC]^high^ slow progressors and an inflammation-dominant profile in [FRC/TLC]^high^ rapid progressors. These proteomic patterns point to divergent pathobiological pathways in pre-COPD: [TLC]^high^ individuals exhibit a systemic antioxidant and repair-skewed environment consistent with restrained inflammation and preserved function, whereas [FRC/TLC]^high^ individuals show a predominantly airway-centered inflammatory and remodeling response. This contrast aligns with their clinical and radiographic profiles and suggests that early disease trajectories may be shaped less by the amount of injury than by the quality of the host response. If validated longitudinally, these findings could refine risk stratification, guide biomarker discovery, and motivate phenotype-tailored interventions targeting systemic repair vs airway inflammation and remodeling. Integrating lung volume–based phenotyping into future observational and interventional studies—paired with longitudinal validation and, where feasible, tissue level assays—will be critical to refine pre-COPD classification and advance personalized approaches to COPD prevention and treatment.

## COPDGene acknowledgments

The COPDGene study was supported by NHLBI grants U01 HL089897 and U01 HL089856 and by NIH contract 75N92023D00011. The COPDGene study (NCT00608764) has also been supported by the COPD Foundation through contributions made to an Industry Advisory Committee that has included AstraZeneca, Bayer Pharmaceuticals, Boehringer-Ingelheim, Genentech, GlaxoSmithKline, Novartis, Pfizer, and Sunovion. The SomaScan data were funded through R01 HL137995 (Bowler).

The University of California San Francisco (UCSF) institutional review board approved the study protocols. The study was also approved by the COPDGene Ancillary Studies and Publications Committee.

## Supplementary Material

aaoag051_Supplementary_Data

## Data Availability

Clinical and research data along with the definition of variables are available on NIH dbGaP for COPDGene study (phs000179.v6.p2).
