# Supplementary material for: Plasma proteomic profiles of lung volume–based phenotypes in tobacco-exposed individuals without spirometric chronic obstructive pulmonary disease
Source: Ann Am Thorac Soc. 2026 Mar 3;23(7):1033–45. doi: 10.1093/annalsats/aaoag051 (PMC13315777; doi:10.1093/annalsats/aaoag051)

**SUPPLEMENTAL TABLES & FIGURES**

**Title:**

Plasma proteomic profiles of lung volume-based phenotypes in Tobacco-Exposed Individuals Without Spirometric COPD

**Author List:**

Siyang Zeng, MS ^1,2^, Claire Guo, BA ^3^, Katherine A Pratte, PhD ^3^, Gang Luo, PhD ^2^, Russell P Bowler, MD, PhD ^3,4^, Mehrdad Arjomandi, MD ^1^

**Affiliations:**

^1^ University of California, San Francisco, California, USA

^2^ University of Washington, Seattle, Washington, USA

^3^ National Jewish Health Systems, Denver, Colorado, USA

^4^ Cleveland Clinic, Cleveland, Ohio, USA

**Corresponding Author:**

Mehrdad Arjomandi, MD

Division of Pulmonary, Critical Care, Allergy, and Sleep Medicine

University of California, San Francisco

4150 Clement Street, Building 203, Room 3A-128, Mailstop 111-D, San Francisco, CA 94121

TEL (415) 221-4810 x24393

EMAIL mehrdad.arjomandi@ucsf.edu

**TABLE OF CONTENTS**

[**SUPPLEMENTAL TABLES** 3](#_Toc213426893)

[**Table S1** 3](#_Toc213426894)

[**Table S2** 5](#_Toc213426895)

[**SUPPLEMENTAL FIGURES** 6](#_Toc213426896)

[**Figure S1** 6](#_Toc213426897)

[**Figure S2** 7](#_Toc213426898)

[**Figure S3** 9](#_Toc213426899)

[**Figure S4** 11](#_Toc213426900)

[**Figure S5** 13](#_Toc213426901)

[**Figure S6** 15](#_Toc213426902)

[**Figure S7** 17](#_Toc213426903)

[**Figure S8** 19](#_Toc213426904)

[**Figure S9** 21](#_Toc213426905)

[**Figure S10** 23](#_Toc213426906)

[**Figure S11** 25](#_Toc213426907)

[**Figure S12** 27](#_Toc213426908)

[**Figure S13** 29](#_Toc213426909)

# **SUPPLEMENTAL TABLES**

## **Table S1**

**Characteristics of TEPS from COPDGene visit 2 (V2 at 5-year follow-up) participants who did not have visit 3 (V3 at 10 year follow-up) data.**

|  | TEPS with  V3 f/u | TEPS without  V3 f/u | All V2 teps |
| --- | --- | --- | --- |
| **Demographic** |  |  |  |
| V2 participants (n) | 1,232 | 727 | 1,959 |
| Age (years) | 63.2±8.1 | 64.0±8.7 | 63.5±8.3 |
| Female [n (%)] | 663 (53.8%) | 370 (50.9%) | 1,033 (52.7%) |
| Height (cm) | 168.9±9.50 | 169.0±9.8 | 169.0±9.6 |
| BMI (kg∙m^-2^) | 29.3±5.9 | 29.1±6.0 | 29.3±5.9 |
| Current smoker [n (%)] | 417 (33.8%) | 326 (44.8%) | 743 (37.9%) |
| Smoking history (pack-years) | 36.7±20.0 | 38.9±21.8 | 37.5±20.7 |
| **Spirometry** |  |  |  |
| FEV_1_ (L) | 2.70±0.65 | 2.66±0.68 | 2.69±0.66 |
| FEV_1_ (% pred) | 98±12 | 98±12 | 98±12 |
| FVC (L) | 3.47±0.84 | 3.41±0.88 | 3.45±0.85 |
| FVC (% pred) | 96±12 | 96±12 | 96±12 |
| FEV_1_/FVC | 0.78±0.05 | 0.78±0.05 | 0.78±0.05 |
| FEV_1_/FVC (% pred) | 102±6 | 102±6 | 102±6 |
| FEF_25-75%_ (L) | 2.55±0.96 | 2.50±0.97 | 2.53±0.96 |
| Bronchodilator responsiveness by FEV_1_ (mL) | 91±151 | 98±163 | 94±156 |
| Bronchodilator responsiveness by FEV_1_ (%) | 3.90±6.70 | 4.14±6.57 | 3.99±6.66 |
| Bronchodilator responsiveness by FEV_1_ [n (%)] | 97 (7.9%) | 63 (8.7%) | 160 (8.2%) |
| **CT indices** |  |  |  |
| FRC (L) | 2.74±0.63 | 2.80±0.65 | 2.77±0.64 |
| TLC (L) | 5.36±1.26 | 5.29±1.26 | 5.33±1.26 |
| FRC/TLC (%) | 52±9 | 54±8 | 53±9 |
| IC (L) | 2.62±0.89 | 2.49±0.87 | 2.57±0.89 |
| HU≤−950 (%) | 9.13±13.0 | 9.7±13.4 | 9.35±13.2 |
| PRM^emph^ (%) | 0.7±1.3 | 0.8±1.8 | 0.7±1.5 |
| HU≤-856 (%) | 52.8±41.5 | 58.5±44.1 | 54.9±42.5 |
| PRM^airtrapping^ (%) | 9.3±7.1 | 9.8±8.5 | 9.4±7.7 |
| Pi10 (%) | 1.9±0.4 | 2.0±0.4 | 1.9±0.4 |

Footnote: Data are presented as mean ± standard deviation or number (n) and percentage (%) of participants. Reference equations: percent predicted of normal values of spirometry and lung volumes were calculated using NHANES III predicted formulas, respectively. Bronchodilator responsiveness was defined as ≥12% and ≥200mL increase in FEV_1_ after bronchodilators administration. Abbreviations: BMI= body mass index; FEV_1_= forced expiratory volume in 1 second; FVC=forced vital capacity; FEF_25-75_= maximum airflow at mid-lung volume; IC= inspiratory capacity; TLC= total lung capacity; FRC= functional residual capacity; Pi10= the mean for the square root of wall area of a hypothetical airway with 10mm internal perimeter; PRM^EMPH^= parametric response mapping of functional small airway disease as measures of emphysema; PRM^Air trapping^= parametric response mapping of percent air trapping; HU≤−856= percentage of the lung voxels with attenuation ≤−856 Hounsfield Unit on the expiratory CT images; HU≤−950= percentage of the lung voxels with attenuation ≤-950 Hounsfield Units on the inspiratory CT images; TEPS= persons with tobacco exposure and preserved spiromtery; V2 and V3 F/U= visit 2 and visit 3 follow-ups.

## **Table S2**

**Area under the receiver operating curve (AUC) with 95% confidence interval of models distinguishing lung volume-based phenotypes using difference sets of predictors.**

|  | **AUC for distinguishing between lung volume-based phenotypes** | | |
| --- | --- | --- | --- |
| **Predictors** | **[TLC]^high^ vs.**  **[FRC/TLC]^high^** | **[TLC]^high^ vs.**  **Low-COPD-risk** | **[FRC/TLC]^high^ vs.**  **Low-COPD-risk** |
| **Covariates only** | 0.73 (0.70 - 0.76) | 0.65 (0.62 - 0.68) | 0.59 (0.55 - 0.62) |
| **Differentially expressed proteins only** | 0.83 (0.80 - 0.85) | 0.73 (0.70 - 0.76) | 0.63 (0.60 - 0.66) |
| **Covariates + differentially expressed proteins** | 0.87 (0.85 - 0.89) | 0.76 (0.73 - 0.78) | 0.64 (0.61 - 0.67) |

Footnote: Area under the receiver operating curve (AUC) with 95% confidence interval for models distinguishing lung volume-based phenotypes using covariates and differentially expressed protein sets corresponding to each phenotype comparison. Covariates included age, sex, height, weight, smoking status, smoking burden, total leukocyte count, platelet count, and FEV_1_ percent predicted. Abbreviations: TLC=total lung capacity; FRC=functional residual capacity.

# **SUPPLEMENTAL FIGURES**

## **Figure S1**

**Workflow for the analysis.** Abbreviations: FDR= false discovery rate.


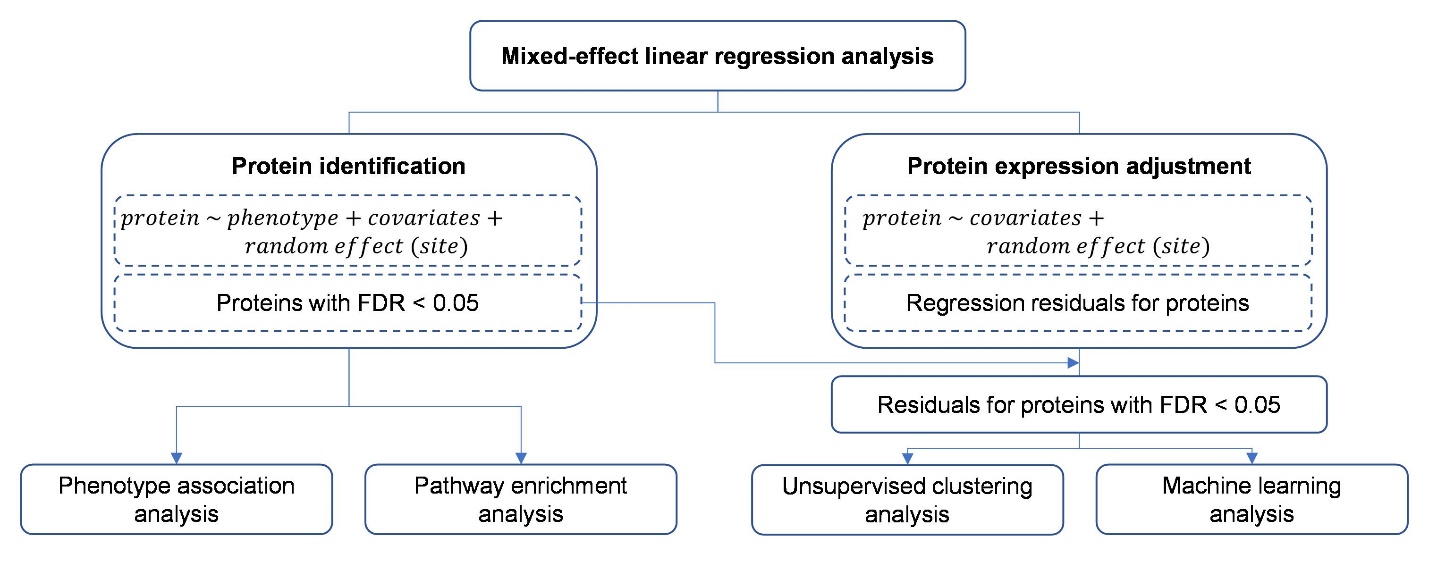


## **Figure S2**

**Spirometric progression of TEPS after 5 years of follow-up from V2 to V3 stratified by lung volumes.** Bar plots show the distribution of COPD GOLD stage at V3 (10-year follow-up) visit with strata of lung volumes generated from V2 (5-year follow-up). The lung volumes were adjusted for age, sex, height, and weight. Horizontal bars indicate statistically significant comparisons of the percentages between the high tertile group in each lung volume stratification with the low tertile group within that stratification using one-way analysis of variance with Tukey Kramer post hoc test. The symbols on the bars indicate statistically significant comparisons of the likelihood of progressing to the corresponding GOLD stages between the high tertiles of different lung volume stratifications using mixed-effect logistic modeling with adjustment for age, sex, height, weight, smoking status (current versus former), and smoking burden (pack-years). GOLD stage 4 was omitted because no participants developed GOLD stage 4. Abbreviations: TEPS= persons with tobacco exposure and preserved spirometry; COPD= Chronic Obstructive Pulmonary Disease; GOLD= Global Initiative for Chronic Obstructive Lung Disease; TLC=total lung capacity; FRC=functional residual capacity.

**
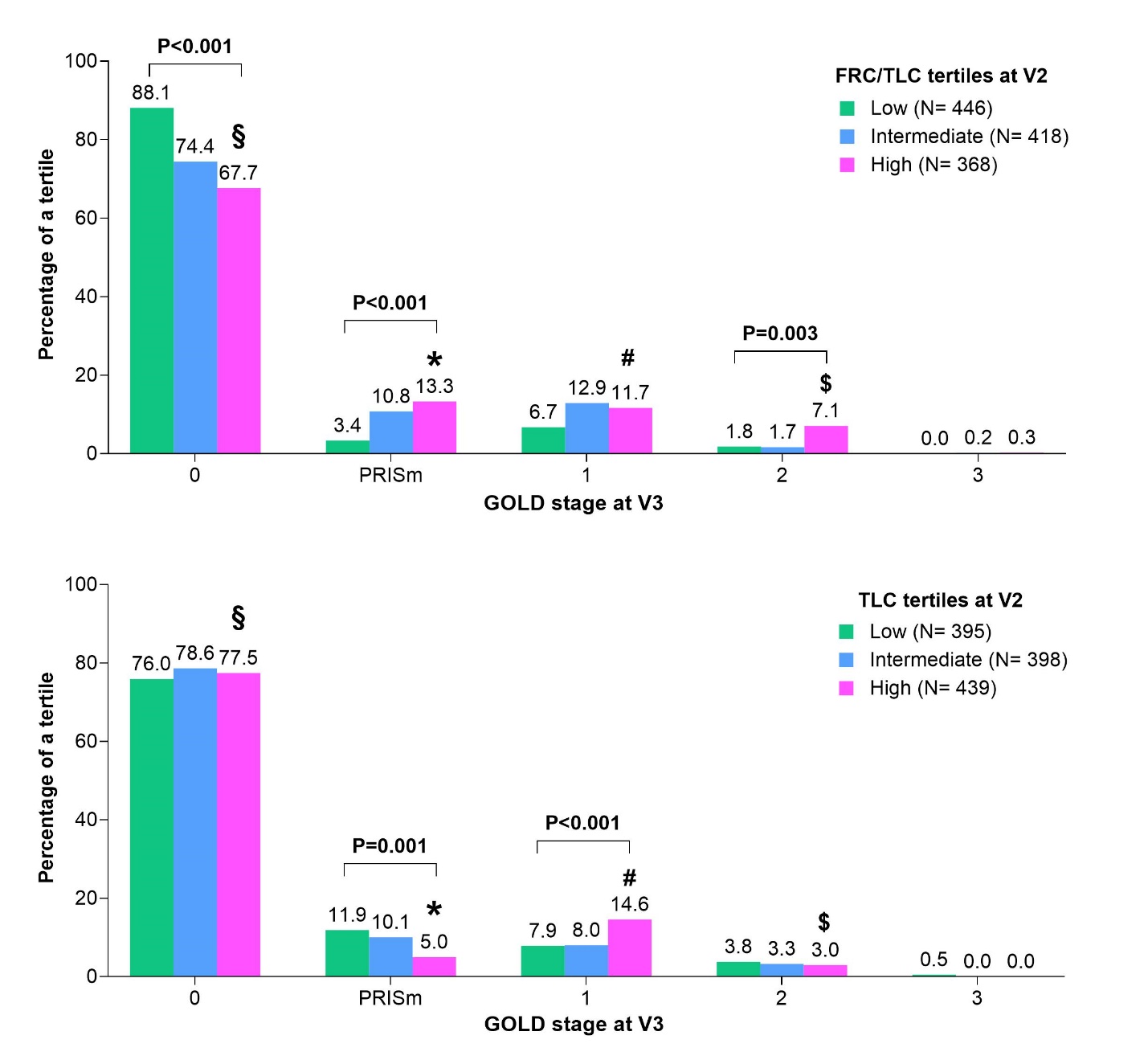
**

## **Figure S3**

**Baseline radiographic features among TEPS stratified by lung volume phenotypes.** The points and the bars represent coefficients and 95% confidence intervals from adjusted regression models of radiographical features for the phenotypes [FRC/TLC]^high^, [FRC/TLC]^high^ & [TLC]^high^, [TLC]^high^, low-COPD-risk (reference group). Abbreviations: TEPS= persons with tobacco exposure and preserved spirometry; TLC=total lung capacity; FRC=functional residual capacity; HU≤-950= percentage of lung voxels with attenuation ≤-950HU on inspiratory CT images; HU≤-856= percentage of lung voxels with attenuation ≤-856HU on the expiratory CT images; Pi10= mean square root of wall area of a hypothetical airway with 10mm internal perimeter); PRM^EMPH^= parametric response mapping for emphysema; PRM^Air trapping^= PRM for air trapping.


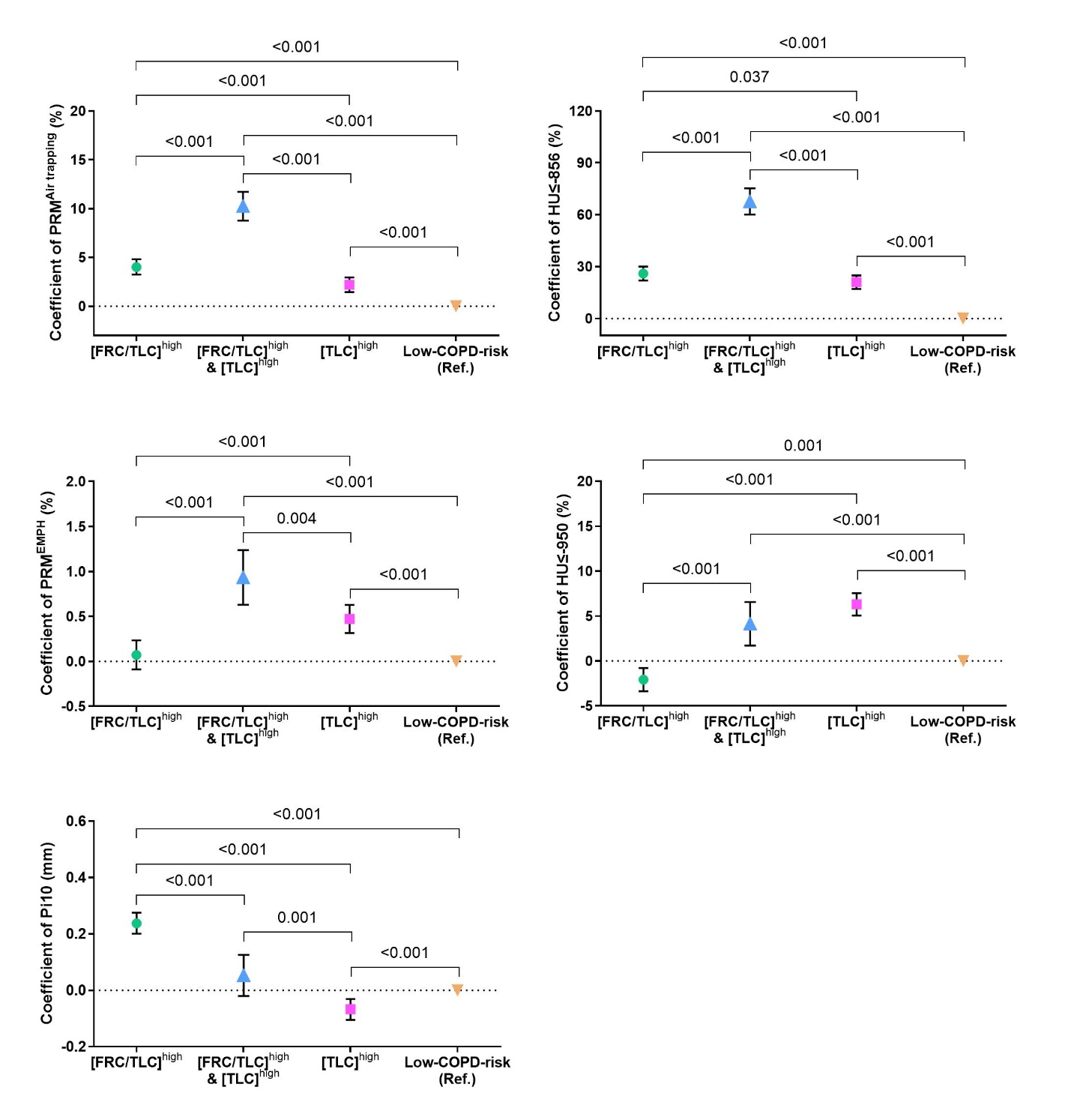


## **Figure S4**

**Baseline clinical characteristics among TEPS stratified by lung volume phenotypes.** The points and the bars represent coefficients and 95% confidence intervals from adjusted regression models of clinical characteristics for the phenotypes [FRC/TLC]^high^, [FRC/TLC]^high^ & [TLC]^high^, [TLC]^high^, low-COPD-risk (reference group). Abbreviations: TEPS= persons with tobacco exposure and preserved spirometry; TLC=total lung capacity; FRC=functional residual capacity; FEV_1_=forced expiratory volume in 1 second; 6MWD= 6-minute walk distance test; mMRC= modified Medical Research Council; SGRQ= St George's Respiratory Questionnaire.

**
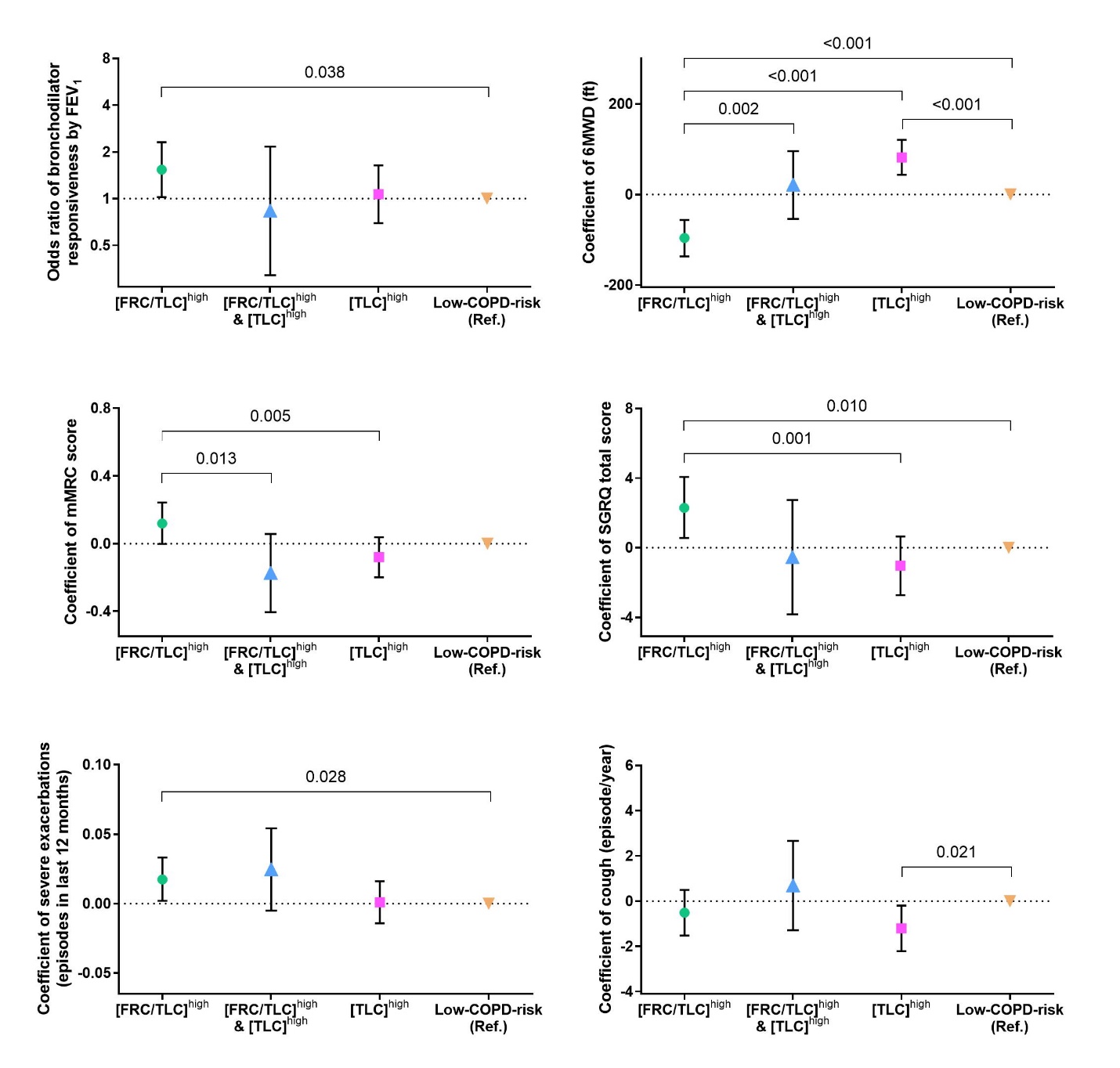
**

## **Figure S5**

**Sensitivity analysis of sets of differentially expressed proteins found in the comparisons of proteomic profiles between the phenotypes.** Differentially expressed proteins found in the comparison between the phenotypes were matched to (**A**) proteins found when the comparison was limited within participants who did not developed PRISm at visit 3 and (**B**) proteins found when the comparison was perform without adjustment to FEV_1_ % predictive. Results were presented using Venn diagrams with numbers indicating the number of distinct and overlapped proteins. Abbreviations: TLC=total lung capacity; FRC=functional residual capacity; FEV_1_=forced expiratory volume in 1 second.

**
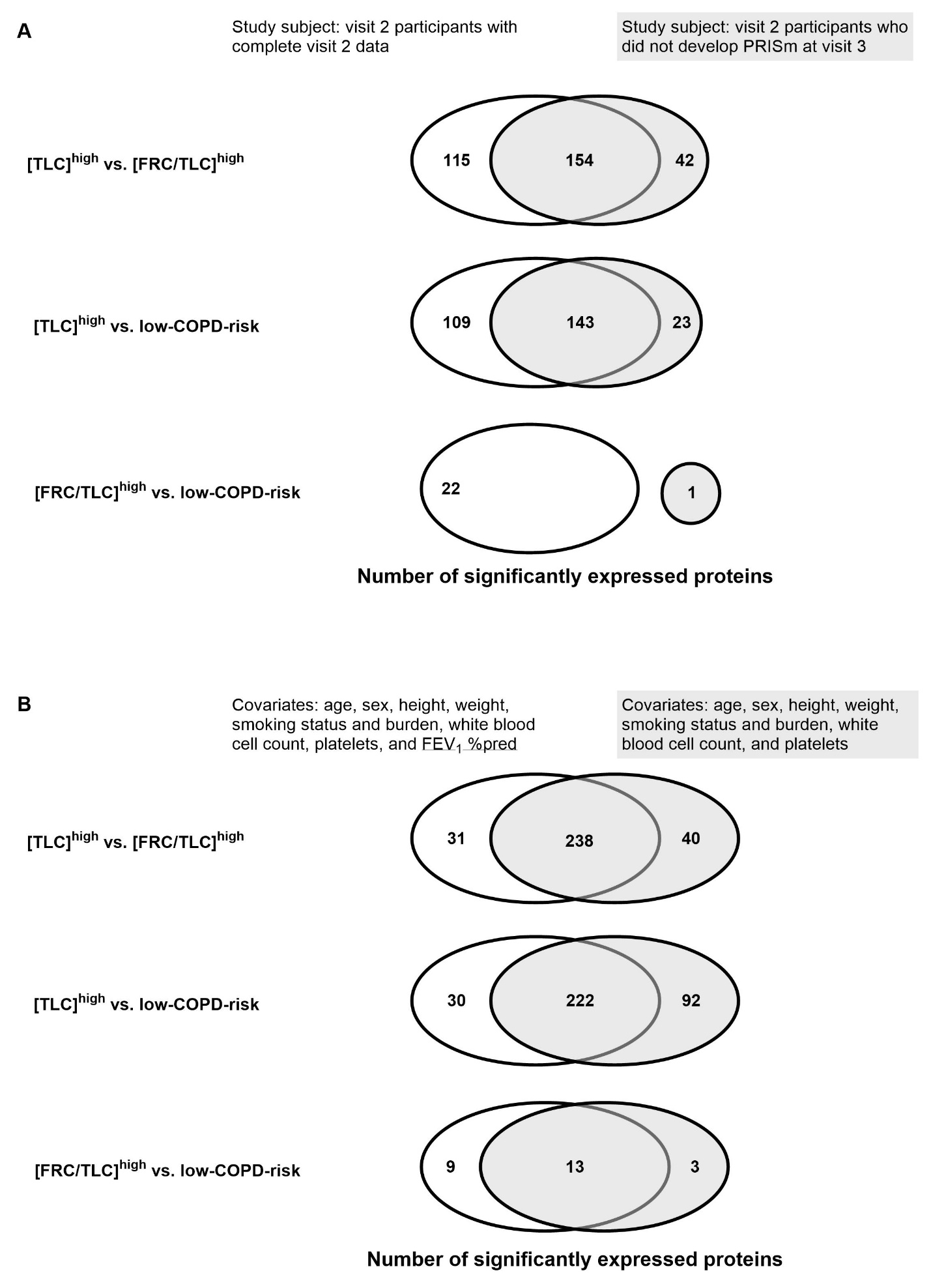
**

## **Figure S6**

**Network graph of the pathways for phenotypes [TLC]^high^ vs. [FRC/TLC]^high^**. The enriched pathways for phenotypes [TLC]^high^ (n=564) vs. [FRC/TLC]^high^ (n=564) were numbered by their fold enrichment values in descending order. Hierarchical clustering was applied to the pathways based on kappa statistics about the differentially expressed proteins involved in each pathway. The resulted clusters of pathways were displayed as a network graph with the cluster number and the names of the pathways provided in text boxes near the clusters. Different clusters and their corresponding text boxes are distinguished by colors. Each node is an enriched pathway. Size of a node is proportional to the number of differentially expressed proteins involved in the pathway. An edge between two nodes indicates that the kappa statistic between the two pathways were greater than 0.35. Abbreviations: [TLC]^high^= TEPS high TLC but not high FRC/TLC; [FRC/TLC]^high^= TEPS with high FRC/TLC but not high TLC; FDR= false discovery rate.

**
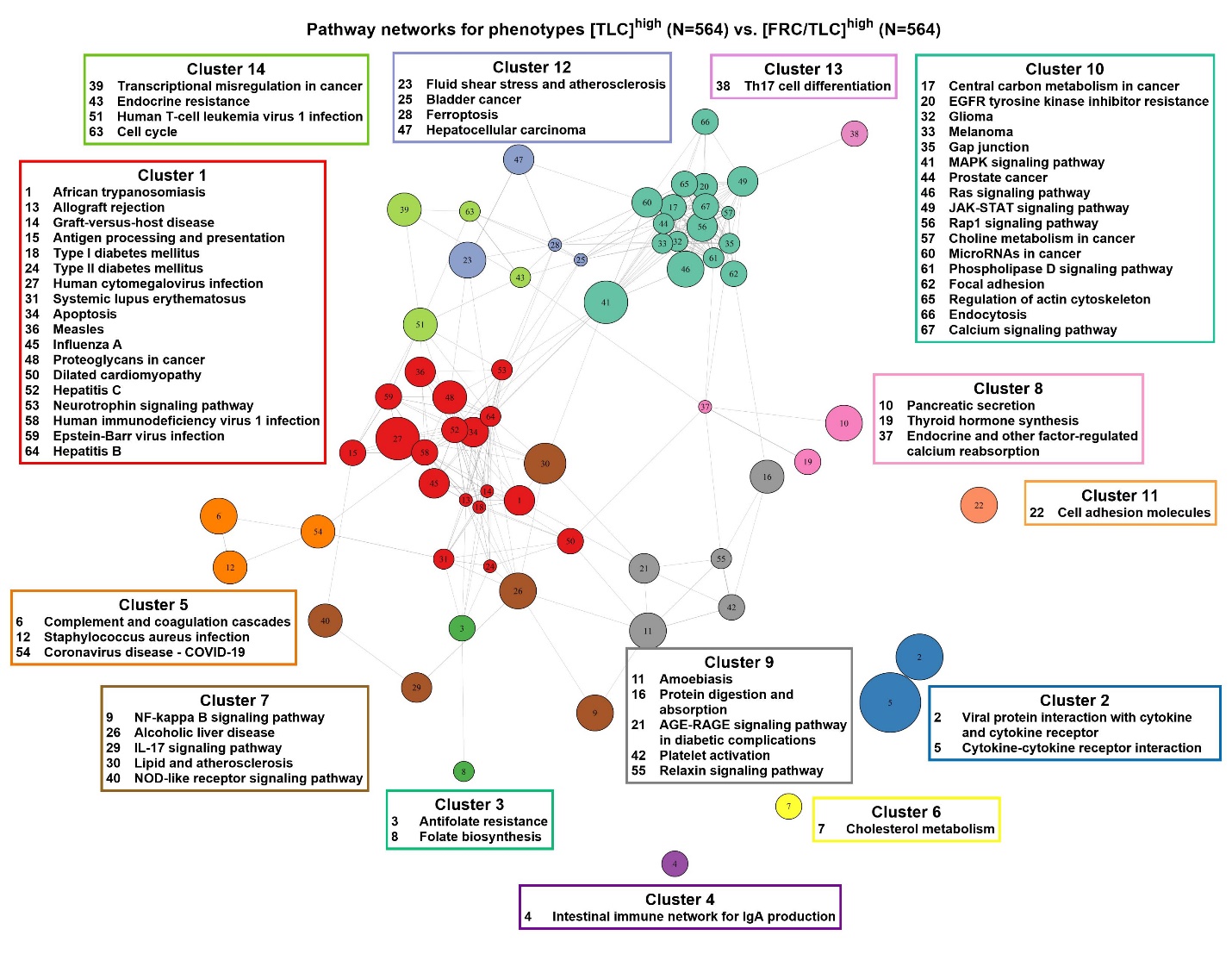
**

## **Figure S7**

**Phenotype association and machine learning results for phenotypes [TLC]^high^ vs. low-COPD-risk.** The difference in plasma proteomics data between [TLC]^high^ (n=564) vs. low-COPD-risk (n=742) was examined using mixed-effect linear regression modeling with adjustment for covariates (age, sex, height, weight, smoking status [current vs. former], smoking burden [pack-years], FEV_1_ % predicted, white blood cell count, platelet count, and random effect of study cite). Differentially expressed proteins for the phenotypes were determined by false discovery rate (FDR) <0.05. Machine learning analysis was performed to evaluate and ranked the differentially expressed proteins for their “feature importance” in distinguishing the phenotypes. **(A)** Heatmap of unsupervised clustering of adjusted expressions of the differentially expressed proteins for [TLC]^high^ vs. low-COPD-risk. The heatmap was horizontally divided into 2 sections by using 2-means clustering before hierarchical clustering on the rows and column. **(B)** Volcano plot for the FDR and β coefficients of all 4,979 proteins compared between [TLC]^high^ and low-COPD-risk. Differentially expressed proteins were marked in blue. Differentially expressed proteins that were ranked top 20 by the machine learning analysis are labeled with protein symbols. **(C)** Bar plots for the “feature importance” of the ranked top 20 differentially expressed proteins for distinguishing between the phenotypes [TLC]^high^ and low-COPD-risk. Abbreviations: TLC=total lung capacity; COPD= Chronic Obstructive Pulmonary Disease; FEV_1_=forced expiratory volume in 1 second; FDR, false discovery rate.


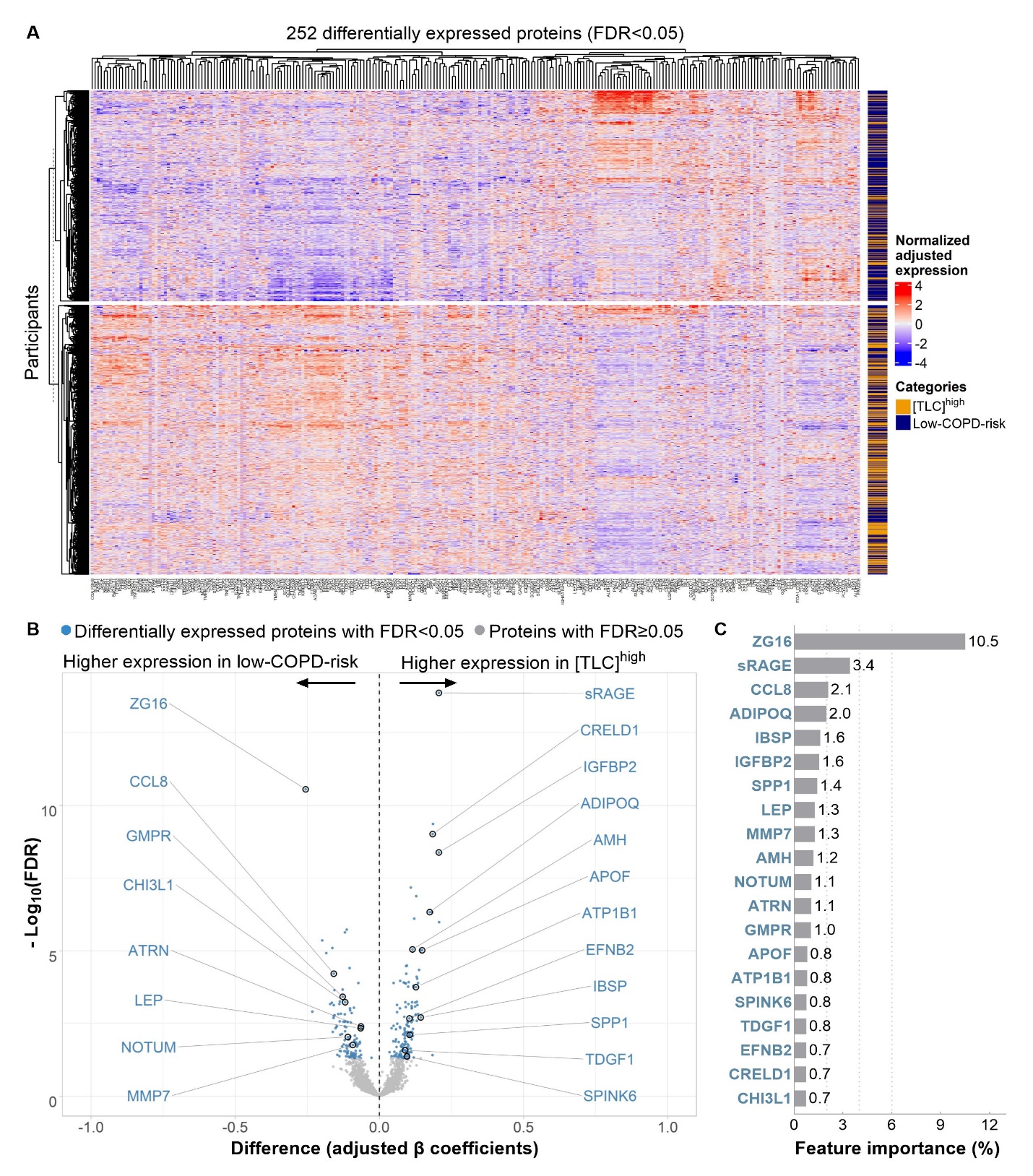


## **Figure S8**

**Phenotype association and machine learning results for phenotypes [FRC/TLC]^high^ vs. low-COPD-risk.** The difference in plasma proteomics data between [FRC/TLC]^high^ (n=564) vs. low-COPD-risk (n=742) was examined using mixed-effect linear regression modeling with adjustment for covariates (age, sex, height, weight, smoking status [current vs. former], smoking burden [pack-years], FEV_1_ % predicted, white blood cell count, platelet count, and random effect of study cite). Differentially expressed proteins for the phenotypes were determined by false discovery rate (FDR) <0.05. Machine learning analysis was performed to evaluate and ranked the differentially expressed proteins for their “feature importance” in distinguishing the phenotypes. **(A)** Heatmap of unsupervised clustering of adjusted expressions of the differentially expressed proteins for [FRC/TLC]^high^ vs. low-COPD-risk. The heatmap was horizontally divided into 2 sections by using 2-means clustering before hierarchical clustering on the rows and column. **(B)** Volcano plot for the FDR and β coefficients of all 4,979 proteins compared between [FRC/TLC]^high^ and low-COPD-risk. Differentially expressed proteins were marked in blue and labeled with protein symbols. **(C)** Bar plots for the “feature importance” of the differentially expressed proteins for distinguishing between the phenotypes [FRC/TLC]^high^ and low-COPD-risk. Abbreviations: FRC=functional residual capacity; TLC=total lung capacity; COPD= Chronic Obstructive Pulmonary Disease; FEV_1_=forced expiratory volume in 1 second; FDR, false discovery rate.

**
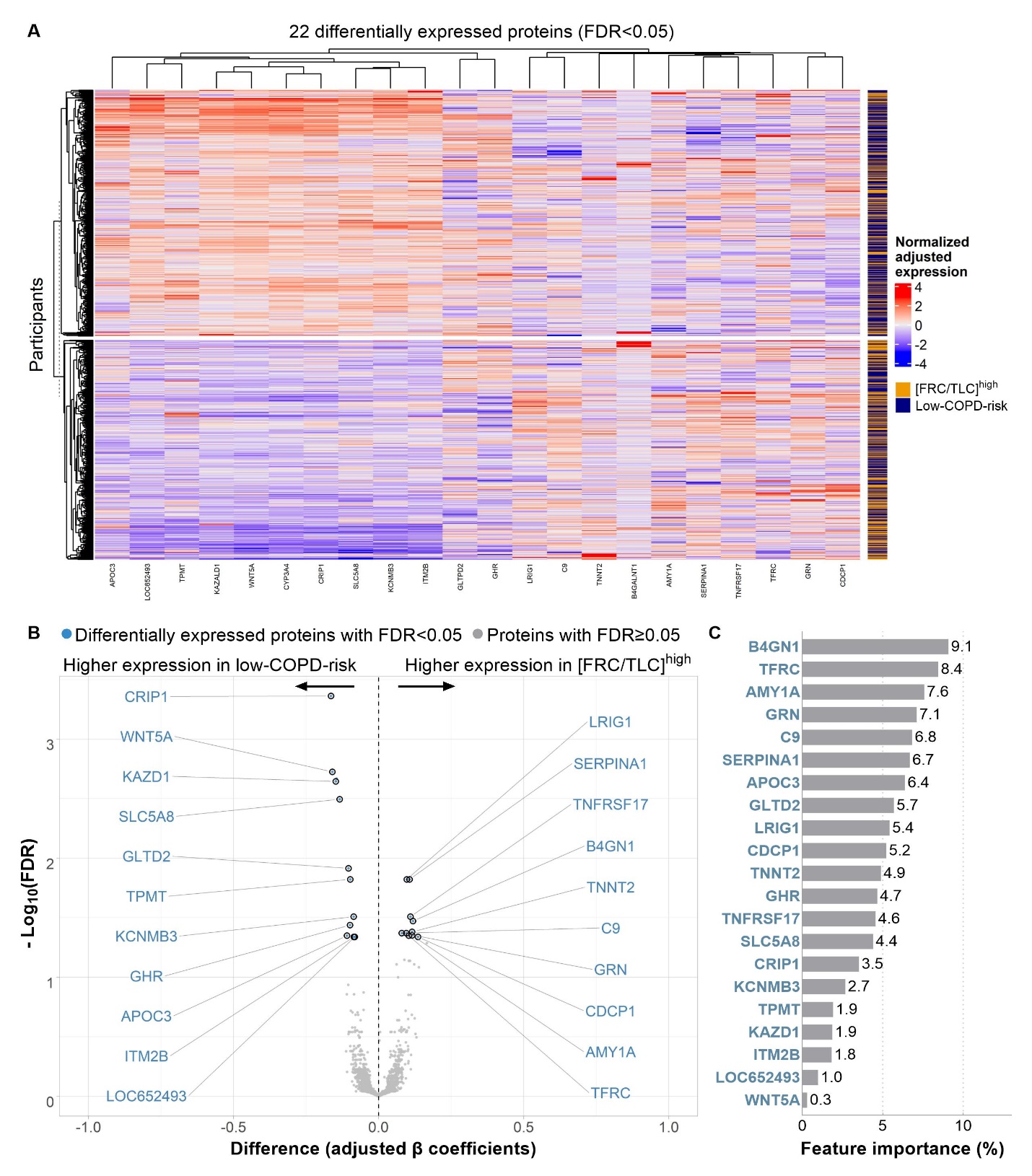
**

## **Figure S9**

**Bubble chart of pathway enrichment results for phenotypes [TLC]^high^ vs. low-COPD-risk.** Hierarchical clustering was applied to the enriched pathways for phenotypes [TLC]^high^ (n=564) vs. low-COPD-risk (n=742) based on kappa statistics about the differentially expressed proteins involved in each pathway. The x-axis corresponds to fold enrichment values, the rate of percentage of differentially expressed proteins belonging to the pathway over the percentage of the pathway’s proteins in the background. The y-axis lists the names of the top 10 pathways with the highest fold enrichment values in each cluster. Each panel with a numbered band on the right side denotes a cluster of pathways. The size of the bubble indicates the number of differentially expressed proteins involved in the given pathway. Color indicates the Bonferroni-adjusted P value of the enrichment by hypergeometric tests; the more it shifts to red, the more significantly the pathway is enriched. Abbreviations: TLC=total lung capacity; COPD= Chronic Obstructive Pulmonary Disease; FDR, false discovery rate.

**
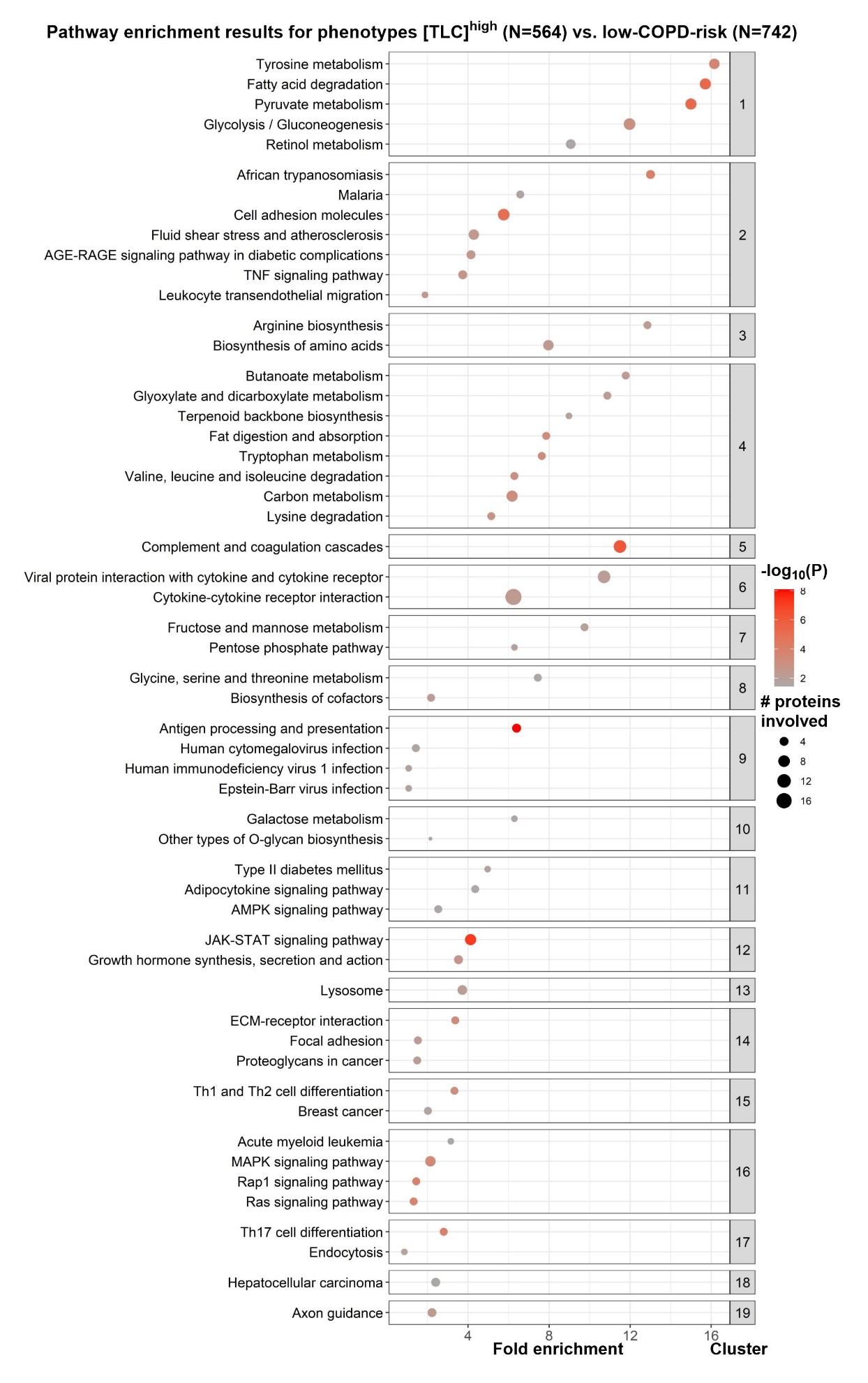
**

## **Figure S10**

**Network graph of the pathways for phenotypes [TLC]^high^ vs. low-COPD-risk**. The enriched pathways for phenotypes [TLC]^high^ (n=564) vs. low-COPD-risk (n=742) were numbered by their fold enrichment values in descending order. Hierarchical clustering was applied to the pathways based on kappa statistics about the differentially expressed proteins involved in each pathway. The resulted clusters of pathways were displayed as a network graph with the cluster number and the names of the pathways provided in text boxes near the clusters. Different clusters and their corresponding text boxes are distinguished by colors. Each node is an enriched pathway. Size of a node is proportional to the number of differentially expressed proteins involved in the pathway. An edge between two nodes indicates that the kappa statistic between the two pathways were greater than 0.35. Abbreviations: TLC=total lung capacity; COPD= Chronic Obstructive Pulmonary Disease; FDR, false discovery rate.

**
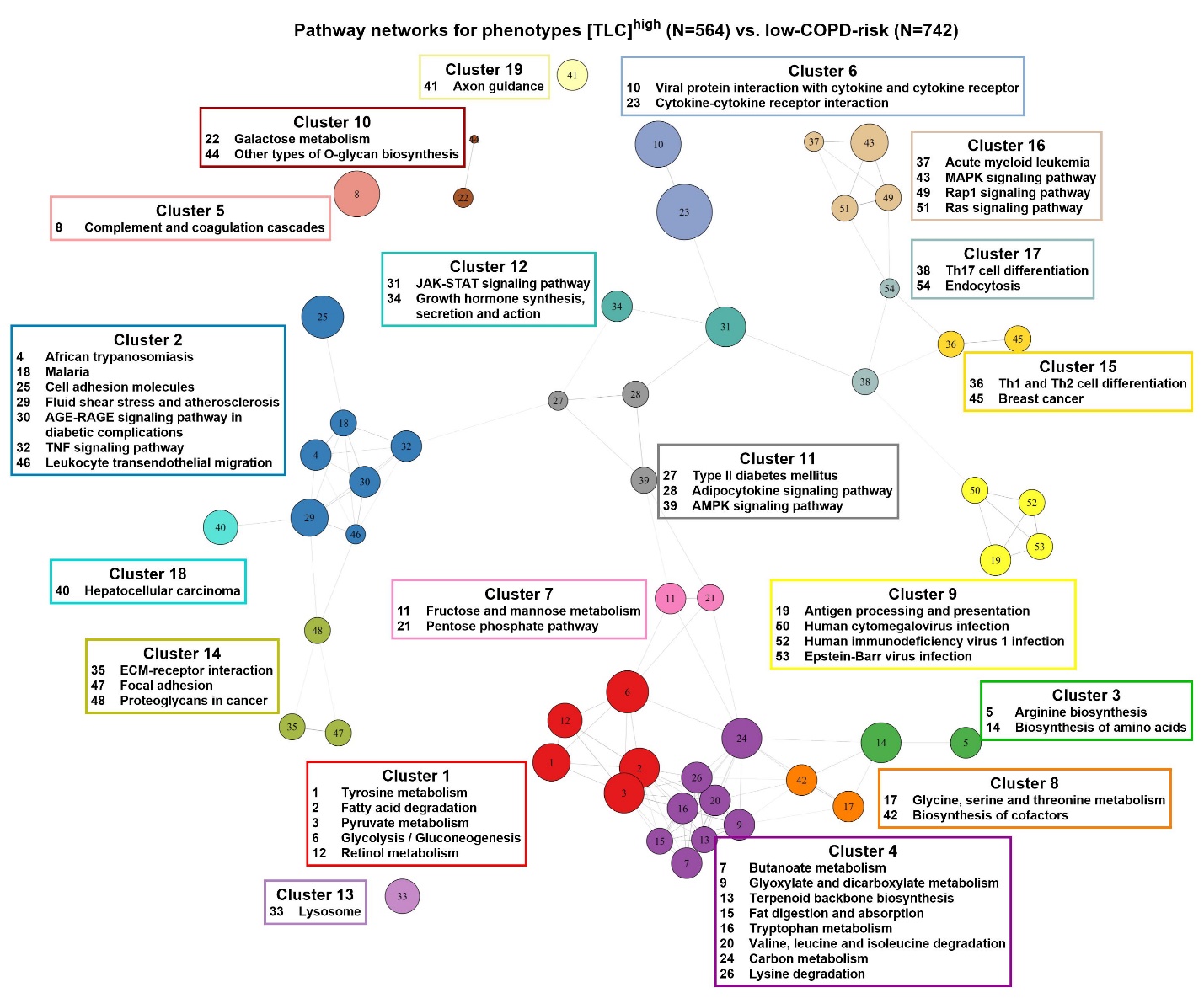
**

## **Figure S11**

**Phenotype association and machine learning results for phenotypes [FRC/TLC]^high^ vs. low-COPD-risk at FDR <0.20.** The difference in plasma proteomics data between [FRC/TLC]^high^ (n=564) vs. low-COPD-risk (n=742) was examined using mixed-effect linear regression modeling with adjustment for covariates (age, sex, height, weight, smoking status [current vs. former], smoking burden [pack-years], FEV_1_ % predicted, white blood cell count, platelet count, and random effect of study cite). Differentially expressed proteins for the phenotypes were determined by false discovery rate (FDR) <0.20. Machine learning analysis was performed to evaluate and ranked the differentially expressed proteins for their “feature importance” in distinguishing the phenotypes. **(A)** Heatmap of unsupervised clustering of adjusted expressions of the differentially expressed proteins for [FRC/TLC]^high^ vs. low-COPD-risk. The heatmap was horizontally divided into 2 sections by using 2-means clustering before hierarchical clustering on the rows and column. **(B)** Volcano plot for the FDR and β coefficients of all 4,979 proteins compared between [FRC/TLC]^high^ and low-COPD-risk. Differentially expressed proteins were marked in blue and labeled with protein symbols. **(C)** Bar plots for the “feature importance” of the differentially expressed proteins for distinguishing between the phenotypes [FRC/TLC]^high^ and low-COPD-risk. Abbreviations: FRC=functional residual capacity; TLC=total lung capacity; COPD= Chronic Obstructive Pulmonary Disease; FEV_1_=forced expiratory volume in 1 second; FDR, false discovery rate.

**
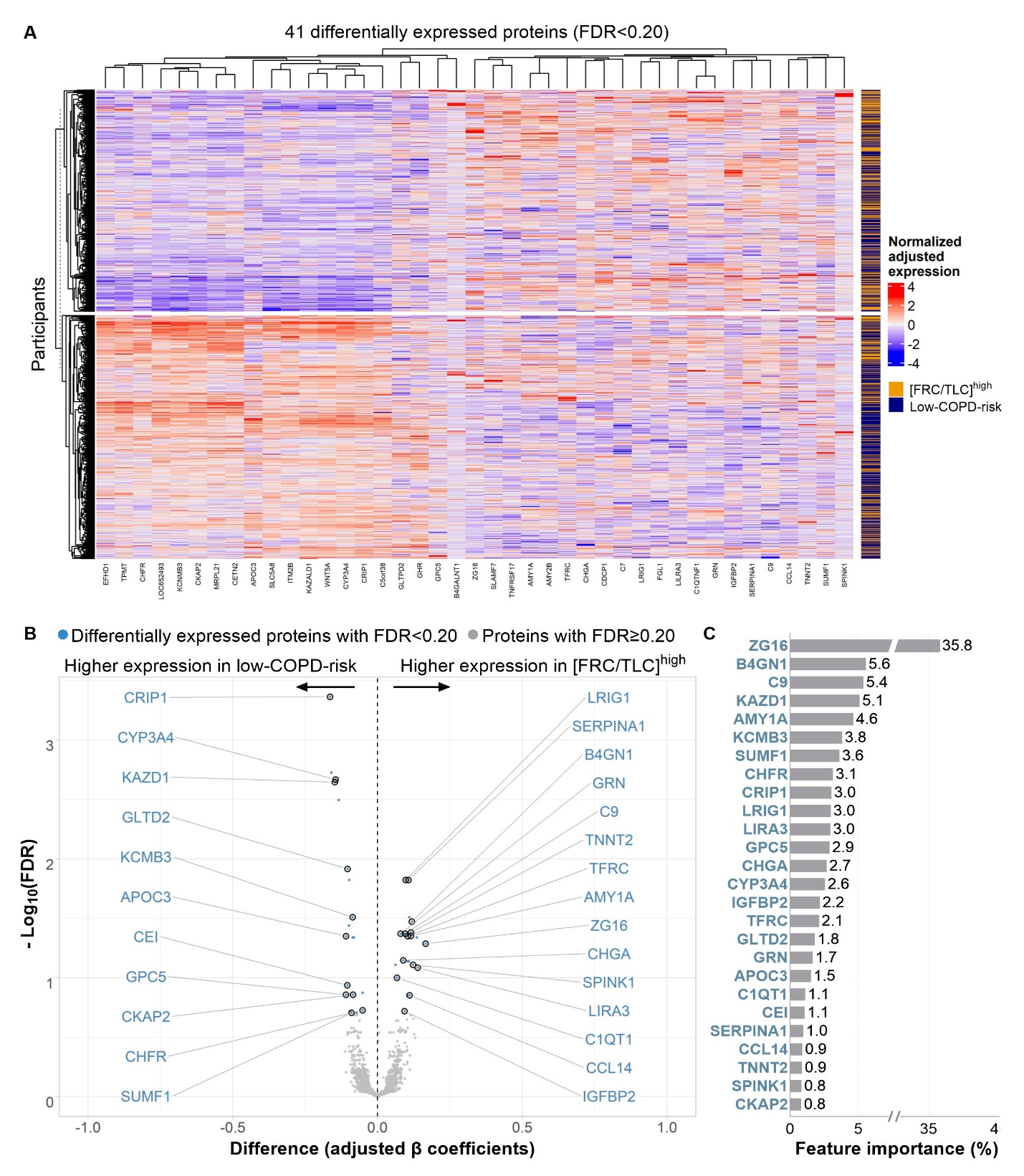
**

## **Figure S12**

**Bubble chart of pathway enrichment results for phenotypes [FRC/TLC]^high^ vs. low-COPD-risk.** Hierarchical clustering was applied to the enriched pathways for phenotypes [FRC/TLC]^high^ (n=564) vs. low-COPD-risk (n=742) based on kappa statistics about the differentially expressed proteins (FDR<0.05 or FDR<0.20) involved in each pathway. The x-axis corresponds to fold enrichment values, the rate of percentage of differentially expressed proteins belonging to the pathway over the percentage of the pathway’s proteins in the background. The y-axis lists the names of the top 10 pathways with the highest fold enrichment values in each cluster. Each panel with a numbered band on the right side denotes a cluster of pathways. The size of the bubble indicates the number of differentially expressed proteins involved in the given pathway. Color indicates the Bonferroni-adjusted P value of the enrichment by hypergeometric tests; the more it shifts to red, the more significantly the pathway is enriched. Abbreviations: FRC=functional residual capacity; TLC=total lung capacity; COPD= Chronic Obstructive Pulmonary Disease; FDR, false discovery rate.


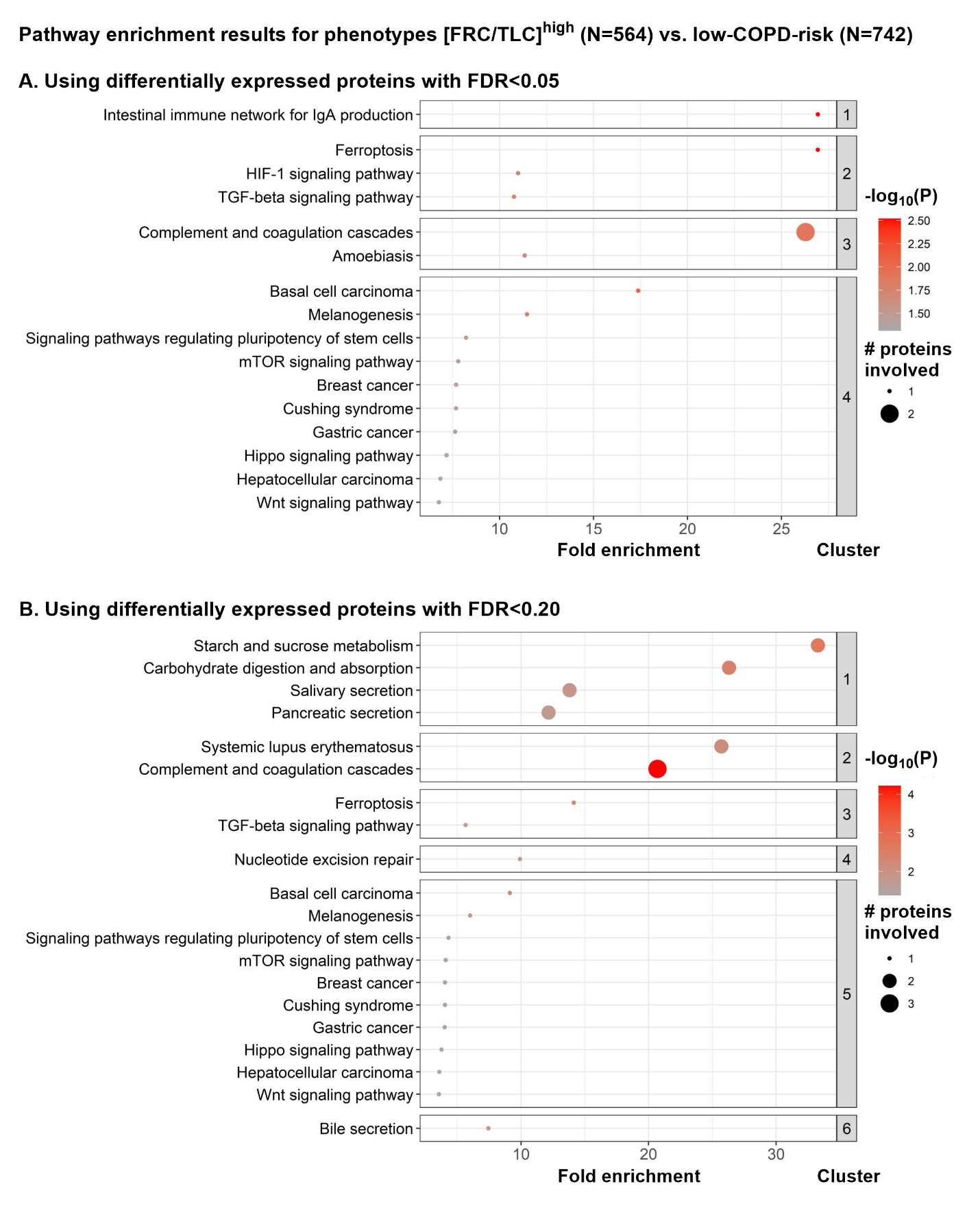


## **Figure S13**

**Network graph of the pathways for phenotypes [FRC/TLC]^high^ vs. low-COPD-risk**. The enriched pathways for phenotypes [FRC/TLC]^high^ (n=564) vs. low-COPD-risk (n=742) were numbered by their fold enrichment values in descending order. Hierarchical clustering was applied to the pathways based on kappa statistics about the differentially expressed proteins (FDR<0.05 or FDR<0.20) involved in each pathway. The resulted clusters of pathways were displayed as a network graph with the cluster number and the names of the pathways provided in text boxes near the clusters. Different clusters and their corresponding text boxes are distinguished by colors. Each node is an enriched pathway. Size of a node is proportional to the number of differentially expressed proteins involved in the pathway. An edge between two nodes indicates that the kappa statistic between the two pathways were greater than 0.35. Abbreviations: FRC=functional residual capacity; TLC=total lung capacity; COPD= Chronic Obstructive Pulmonary Disease; FDR, false discovery rate.


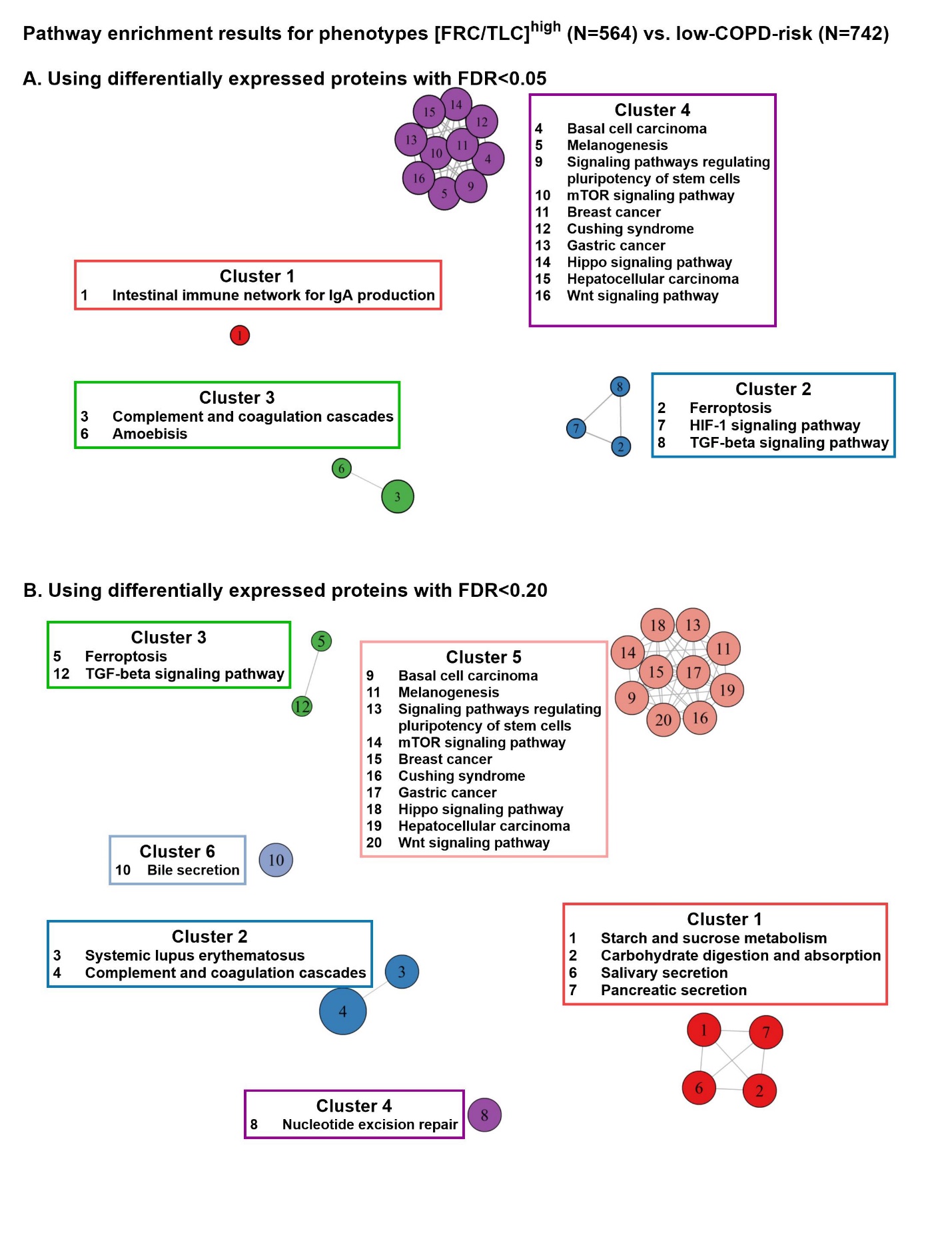

Supplement: aaoag051_Supplementary_Data [file aaoag051_supplementary_data.zip › Supp_Tabs_Figs_LV_COPDGProt_102725_v8.2.docx]
