# Supplementary material for: Plasma proteomic profiles of lung volume–based phenotypes in tobacco-exposed individuals without spirometric chronic obstructive pulmonary disease
Source: Ann Am Thorac Soc. 2026 Mar 3;23(7):1033–45. doi: 10.1093/annalsats/aaoag051 (PMC13315777; doi:10.1093/annalsats/aaoag051)
Supplement: aaoag051_Supplementary_Data [file aaoag051_supplementary_data.zip › SuppMethods_LV_COPDGProt_102325_v7.1.docx]

**SUPPLEMENTAL METHODS**

**Title:**

Plasma proteomic profiles of lung volume-based phenotypes in Tobacco-Exposed Individuals Without Spirometric COPD

**Author List:**

Siyang Zeng, MS ^1,2^, Claire Guo, BA ^3^, Katherine A Pratte, PhD ^3^, Gang Luo, PhD ^2^, Russell P Bowler, MD, PhD ^3,4^, Mehrdad Arjomandi, MD ^1^

**Affiliations:**

^1^ University of California, San Francisco, California, USA

^2^ University of Washington, Seattle, Washington, USA

^3^ National Jewish Health Systems, Denver, Colorado, USA

^4^ Cleveland Clinic, Cleveland, Ohio, USA

**Corresponding Author:**

**Mehrdad Arjomandi, MD**

Division of Pulmonary, Critical Care, Allergy, and Sleep Medicine

University of California, San Francisco

4150 Clement Street, Building 203, Room 3A-128, Mailstop 111-D, San Francisco, CA 94121

TEL (415) 221-4810 x24393

EMAIL mehrdad.arjomandi@ucsf.edu

**Study design**

*COPDGene study design*

The COPDGene study is a U.S.-based multicenter observational prospective study designed to identify genetic factors associated with COPD. From 2007 to 2010, COPDGene (ClinialTrials.gov Identifier: NCT00608764) enrolled 10,263 people with history of current and former smoking with or without COPD.^1^ The COPDGene study inclusion criteria were: non-Hispanic White or African-American, age 45 to 80 years, and history of current or former smoking (≥10 pack-years). Participants reporting a medical diagnosis of active lung diseases other than asthma, emphysema, chronic bronchitis, or COPD were excluded (e.g., lung cancer). The goals of the COPDGene study have been to characterize phenotypes of people with history of tobacco smoking using spirometry, chest computerized tomographic (CT) scans (at full inspiration [TLC] and normal exhalation [FRC]), and medical history and questionnaires regarding respiratory symptoms, and to perform genome-wide association studies (GWAS). Local IRB approvals to enroll participants in COPDGene study were obtained and all participants provided informed consent to participate in the study.

The COPDGene study involved two follow-up visits occurring at 5 years (V2) and 10 years (V3) from the initial visit (V1) with progressively fewer participants partaking in the subsequent visits. Questionnaires, CT imaging, and blood samples were obtained at all three visits. Proteomics using SomaScan (version 4.0; 4,979 aptamers, mapping to 4,776 unique human proteins) was performed by SomaLogic, Inc. on plasma samples from second visit (V2) using aptamer-based SomaScan platforms version 4.0 that included 4,979 human SOMAmers mapping to 4,776 unique human proteins.

*Current study design*

SomaScan version 4.0 proteomics data was only available from the second visit (V2) of COPDGene participants and not from their initial visit (V1). Thus, the current analysis focused on participants who contributed to the second visit (V2) data. “Baseline” (V2) data was derived from all participants who had a history of smoking tobacco, had undergone pre- and post-bronchodilator spirometry (two inhalations of albuterol 90 μg per inhalation with repeat spirometry 15 minutes later), and had chest CT imaging with radiographic lung volumes (TLC and FRC) measurements. For the current study, TEPS participants defined as those with history of current and former ≥10 pack-years smoking with preserved spirometry (forced expiratory volume in 1 second to forced vital capacity ratio or FEV_1_/FVC ≥0.70 after bronchodilator administration)^2^ at V2 were included. TEPS participants with preserved ratio and impaired spirometry (post-bronchodilator FEV_1_/FVC ≥0.70 but FEV_1_ ≤80% of predicted; PRISm) were not included in the analyses for the current study. Follow-up data were derived from participants who had completed their 10-year follow-up visits (V3).

Lung volume-based phenotyping of TEPS was done using V2 CT-measured TLC and FRC, as shown in **Figure 1** and described previously.^3^ CT-measured TLC and FRC/TLC were adjusted for covariates (age, sex, height, and weight) and then stratified by tertiles to obtain low, intermediate, and high strata.^3^ Mutually exclusive lung volume strata consisting of TEPS with (1) high TLC but not high FRC/TLC (termed [TLC]^high^) and (2) high FRC/TLC but not high TLC (termed [FRC/TLC]^high^) were generated.^3,4^ The “baseline” V2 clinical and radiographical characteristics and disease trajectories (V3) of these lung volume-based phenotypes of TEPS from V2 were examined, as was described previously by us for V1 lung volume-based stratification.^3^

**CT Indices of Lung Volumes, Air Trapping, Emphysema, and Small Airways**

The detailed protocol and quality assessment of COPDGene CT scan imaging have been described previously.^1^ Briefly, participants underwent two volumetric chest CT examinations, one at full inspiration (TLC) and one at the end of a relaxed exhalation (FRC). Three manufacturers and 11 different CT scanner models were used in the study including those with 16-detector (1,083 participants), 40-detector (12 participants), 64-detector (1667 participants), and 128-detector (1300 participants) scanners.^5^ Anonymized scans were transferred to a central imaging laboratory for quantitative analysis using a standardized protocol with image reconstruction at sub-millimeter slice thickness with smooth and edge-enhancing algorithms.^1,5,6^

For our study, the following CT-measured parameters were used: CT-measured TLC and FRC and their ratio (FRC/TLC), CT indices of air trapping (including the percent of the lung voxels with attenuation <-856 HU on the expiratory CT images (HU≤-856)^7,8^ and parametric response mapping of air trapping (PRM^Air trapping^)^9,10^), measures of emphysema (including the percent of the lung voxels on inspiratory CT images with attenuation ≤-950HU (HU≤-950) and parametric response mapping of emphysema (PRM^EMPH^)^9,10^), and measures of airway disease (including the mean value for the square root of wall area of a hypothetical airway with 10 mm internal perimeter (Pi10)^11^).

**Pulmonary Function testing**

The detailed protocol and quality assessment of COPDGene lung function measurements have been described previously.^1^ Briefly, spirometry was performed following the American Thoracic Society guidelines and using the EasyOne spirometer (ndd Medical Technologies, Andover, Massachusetts).^12^ Spirometry was performed at baseline before and then repeated after the administration of 180 mcg of inhaled albuterol. Peak expiratory flow (PEF), FVC, FEV_1_, and forced mid-expiratory flow (FEF_25-75%_) were obtained for all participants. The percent predicted values for FEV_1_ and FVC were calculated using the Third National Health and Nutrition Examination Survey (NHANES) reference values.^2^ The ratio of FEV_1_ to FVC (FEV_1_/FVC) was calculated using the absolute measures in liters, and a ratio less than 0.70 at baseline was used to diagnose spirometric COPD, and severity of the disease was graded per the GOLD recommendations.^13^

**SomaScan**

COPDGene plasma samples were analyzed using SomaScan v4.0 (Somalogic, Inc., Boulder), which utilizes a pool of specific aptamers (SOMAmers) that bind to epitopes in the sample matrix, to measure relative abundances of proteins from a sample volume of ~60 µL.^14^ SomaScan v4.0, which measures 4,979 SOMAmers (“5k”), mapping to 4,776 unique human proteins, is performed on a custom Agilent oligonucleotide microarray, generating a Relative Fluorescence Unit (RFU) for each SOMAmer. The SomaScan assay includes a 96-well plate format. Each plate includes replicate controls for a) buffer only, (b) a stock calibrator matching the submitted sample matrix, and (c) quality control (QC) samples of the same matrix to evaluate assay run conditions and normalization parameters. Both 96-well plate identifications (IDs) and Agilent Scanner IDs are tracked and assessed during this process.

The generated data then underwent technical quality assessment using a standardized analysis anchored on the library arrayQualityMetrics. Individual samples were evaluated using three outlier detection methods implemented in arrayQualityMetrics. Samples exhibiting extreme D-statistics and corresponding MA plots were flagged. SomaLogic’s additional outlier flagging for normalization deviations informed the subsequent analysis, and flagged samples were excluded if they failed quality assurance (QA) checks. This included normalization, calibration, and scaling consisted of: (1) within-plate hybridization to control for variability across array signals, (2) median signal normalization to account for technical variability within-run replicates, (3) plate scaling and SOMAmer calibration to address inter-assay variation and batch differences between plates, and (4) adaptive normalization by maximum likelihood within dilution groups to quality control replicates and individual samples. Edge effects and technical variance were minimized.^14^. The adaptive normalization censors the analytes that fall outside the expected range by their maximum likelihood based on the reference population distribution (median and variance) by calculating a scaling factor to maximize the likelihood that a sample’s RFU measurement originates from the reference distribution. All values were log-transformed prior to analysis.^15,16^ Results that did not meet QA standards were removed from further analysis.^14-16^

**Data Management and Statistical Analysis**

We summarized the entire analysis workflow integrating data preprocessing, mixed-effect linear regression analysis, machine learning analysis, principal component analysis, and pathway enrichment analysis in **Figure S1**. Data management, analysis, and visualizations were done in R (version 4.2.2; R Foundation for Statistical Computing, Vienna, Austria). Figures were generated in GraphPad Prism (version 9.0.0; GraphPad Software, San Diego, CA, USA).

Covariates for adjustment were selected based on the factors that are conventionally considered to be important source of confounding in each analysis. For adjustment of lung volume measures, age, sex, height, and weight were included as covariates, as we previously described.^17^ For outcomes analyses, age, sex, height, and weight, as well as smoking status (current versus former), smoking burden (pack-years), and corresponding follow-up time were used as additional factors that could have confounded the analysis of those outcomes, as we previously described.^18^ For analysis of longitudinal changes in outcomes, in addition to the above covariates, corresponding baseline measurements were also included as covariate to account for dissimilar baseline measurements of the outcome of interest. Proteomic analyses were adjusted for age, sex, height, weight, smoking status, and smoking burden, as well as total leukocyte, and platelet count, as recommended by SomaLogic for SomaScan plasma proteomic data on the basis of prior internal quality control.^19,20^ Finally, FEV1 (as percent predicted) was also included as a cofounder to account for FEV_1_ variation in within the normal ranges that may be seen with each specific lung volume indices among TEPS.^3,4,17,21,22^

*Lung volume stratification and longitudinal follow up analysis*

As currently no validated reference values for CT-measured lung volumes are available, regression modeling with adjustment for covariates was applied. We derived adjusted lung volumes by computing the “residual” values from linear regression modeling of the absolute lung volumes over age, sex, height, and weight, which were then used in further analysis.

Regression modeling was done using lung volumes as continuous and categorical variables. Stratification of lung volumes into categorical variables were done based on our previous studies from electronic health records of patients with history of smoking but preserved spirometry (GOLD-0), which showed that about a third of such patients had lung volumes greater than their predicted upper limit of normal values.^23^ Based on those findings, and considering the lack of reference values for CT-measured lung volumes for their stratification as normal and abnormal, we stratified the participants into three equal categories of low-, intermediate-, and high-tertiles using their adjusted lung volumes (i.e., tertiles of FRC, tertiles of TLC, and tertiles of FRC/TLC).

Longitudinal outcome of developing spirometric COPD were assessed at the 10-year follow-up visit (V3) and analyzed with respect to the adjusted lung volumes (TLC, FRC, and FRC/TLC) tertiles using mixed effect logistic modeling. Longitudinal changes in spirometric indices (FEV_1_, FVC, and FEV_1_/FVC) were calculated by subtracting the subsequent visit (V3) values from the “baseline” visit (V2) values and analyzed with respect to the adjusted lung volumes tertiles using mixed effect linear modeling. All models were adjusted for age, sex, height, weight, smoking status (current versus former), smoking burden (pack-years), and years between the visits as fixed effects and study site as the random effect. Additionally, models for spirometric indices were also adjusted for their respected spirometric index baseline value (for example, the model for change in FEV_1_ included baseline FEV_1_ from V2 as a covariate in the model). Distributions of subsequent GOLD stages at V3 with respect to the lung volumes tertiles were summarized and compared using one-way analysis of variance with Tukey Kramer post hoc for multiple-group comparisons.

Because the high tertiles of TLC and FRC/TLC seemed to provide diverging predictive models for change in spirometric indices, we then generated mutually exclusive categories of high TLC but no high FRC/TLC ([TLC]^high^) and high FRC/TLC but no high TLC ([FRC/TLC]^high^), as well as a third category that included those with both high TLC and high FRC/TLC ([FRC/TLC]^high^ & [FRC/TLC]^high^), and compared the baseline characteristics and longitudinal outcomes between these categories.

CT-related mechanistic measures (HU≤-950, PRM^EMPH^, HU≤-856, PRM^Air trapping^, and Pi10) and characteristics and outcomes including bronchodilator responsiveness, exercise capacity (6-Minute Walk Distance (6-MWD) test), symptoms (Modified Medical Research Council Dyspnea Scale (mMRC), cough, and Saint George’s Respiratory Questionnaire (SGRQ)), and self-reported severe exacerbations were examined at baseline and subsequent visits using mixed effect logistic or linear modeling (for binary or continuous dependent variables, respectively) with adjustment for age, sex, height, weight, smoking status (current versus former), and smoking burden (pack-years) as fixed effects and study site as the random effect.

*Proteomics data preprocessing*

After integrating CT, lung function, and proteomics data, we performed the variance stabilization normalization^24^ to the quantitative variables using R package *vsn* (version 3.66.0).^25^ Participants with missing values were omitted from the analysis.

*Protein identification*

We performed three separate analyses to examine the proteomic profiles of the three comparisons between: (1) the two pre-COPD phenotypes of [TLC]^high^ and [FRC/TLC]^high^ (primary analysis), (2) [TLC]^high^ and low-COPD-risk, and (3) [FRC/TLC]^high^ and low-COPD-risk. The plasma proteomics data was examined using mixed-effect linear regression modeling with adjustment for covariates (age, sex, height, weight, smoking status [current vs. former], smoking burden [pack-years], FEV_1_ % predicted, white blood cell count, platelet count, and random effect of study site) as

$protein \sim phenotype \left( HE or AT \right)+age+sex+height+weight+smoking status+smoking burden+WBC+platelets+{FEV}_{1}+random effect (study site)$.

P values were corrected for multiple testing with the Benjamini-Hochberg False Discovery Rate (**FDR**) method. An FDR <0.05 was used to identify differentially expressed proteins. Regression analysis was performed using R package *lme4* (version 1.1-31).^26^

Sensitivity analyses of proteomic profile comparisons were performed in participants who did not developed PRISm in visit 3. Differentially expressed proteins were matched with those found for in analyses using all participants with complete visit 2 data.

*Phenotype association analysis*

Differentially expressed proteins associated with the phenotype were examined by the sign and magnitude of the parameter estimate (adjusted fold-change) using volcano plots.

*Pathway enrichment analysis*

To investigate the potential biological functions of the proteins distinguishing the phenotypes, we performed pathway enrichment analysis via active subnetwork search in protein-protein interaction networks including KEGG and Gene Ontology. Using the differentially expressed proteins, we identified distinct phenotype-associated sets of interacting proteins that form active subnetworks as well as enriched pathways that contain the differentially expressed proteins in the active subnetworks. Specifically, for each active subnetwork, a hypergeometric distribution test was performed using the differentially expressed protein involved in the active subnetwork to determine whether the differentially expressed proteins were enriched (compared to the background pool of proteins, i.e., the proteins used in the experiment). Subnetworks with a Bonferroni-adjusted P<0.05 were considered enriched pathways. A fold enrichment value was calculated for each pathway as the rate of percentage of differentially expressed proteins belonging to the pathway over the percentage of the pathway’s proteins in the background pool of proteins. For each pair of pathways found, a kappa statistics was calculated based on the proteins involved in the pathways. Hierarchical clustering was then performed using the kappa statistics. The optimal number of clusters was determined by the number which yielded the highest average silhouette score. A network plot of the pathways was generated based on the kappa statistics and the clusters. Pathway analysis was performed using R package *PathfindR* (version 2.4.1).^27^

*Protein expression adjustment*

Using the similar mixed-effect linear regression modelling but removing the phenotype, we computed the residual values from the regression model for each protein to be the adjusted protein expressions. The residuals were computed from regression models as

$protein \sim age+sex+height+weight+smoking status+smoking burden+WBC+platelets+{FEV}_{1}+random effect (study site)$.

*Unsupervised clustering analysis*

Adjusted expression for the differentially expressed proteins were z-normalized and then visualized in a heatmap. Unsupervised hierarchical clustering of the heatmap was used to examine the differences between the phenotypes on the basis of dendrograms and adjusted protein expression. Heatmap with hierarchical clustering was performed using R package *ComplexHeatmap* (version 2.14.0).^28^

*Machine learning analysis*

To evaluate how much the proteins contributed to differentiate the phenotypes, we performed machine learning analysis using the significantly expressed proteins identified from the regression analysis, inclusively in one model, to predict the phenotypes. We applied three-, four-, and five-fold nested cross-validation (NCV)^29^ using three widely used machine learning algorithms (XGBoost, Random Forest, and Naïve Bayes). Each NCV consisted of an inner loop to optimize the hyper-parameter(s) and an outer loop to evaluate the performance of the models using the optimal hyper-parameter(s) determined in the corresponding inner loops. For each round of NCV in the outer loop, we calculated the accuracy and area under the receiver operating characteristic curve (AUC). The final performance of a NCV was determined by the averaged AUC from its outer rounds. “Feature importance” was estimated from each model to quantify each protein’s contribution (as a percentage) to outcome prediction.

The NCV was performed using R packages *nestedcv* (version 0.7.8)^30^ and *caret* (version 6.0-93)^31^ with methods as "xgbTree" (XGBoost), “rf” (Random Forest), and “nb” (Naïve Bayes). Those methods were performed using R package *xgboost* (version 1.7.3.1) (XGBoost),^32^ *randomForest* (version 4.7-1.1) (Random Forest),^33^ and *klaR* (version 1.7-3) (Naïve Bayes).^34^

Data management, distribution analysis, and mixed effect regression were done in R (version 4.0.1; R Foundation for Statistical Computing, Vienna, Austria). Figures were generated in GraphPad Prism (version 9.0.0; GraphPad Software, San Diego, CA, USA).

# **REFERENCES**

1. Regan EA, Hokanson JE, Murphy JR, et al. Genetic epidemiology of COPD (COPDGene) study design. COPD 2010;7(1):32–43. DOI: 10.3109/15412550903499522.

2. Hankinson JL, Odencrantz JR, Fedan KB. Spirometric reference values from a sample of the general U.S. population. Am J Respir Crit Care Med 1999;159(1):179–87. (In eng). DOI: 10.1164/ajrccm.159.1.9712108.

3. Zeng S, Luo G, Lynch DA, Bowler RP, Arjomandi M. Lung volumes differentiate the predominance of emphysema versus airway disease phenotype in early COPD: an observational study of the COPDGene cohort. ERJ Open Res 2023;9(5) (In eng). DOI: 10.1183/23120541.00289-2023.

4. Zeng S, Tham A, Bos B, Jin J, Giang B, Arjomandi M. Lung volume indices predict morbidity in smokers with preserved spirometry. Thorax 2019;74(2):114–124. (In eng). DOI: 10.1136/thoraxjnl-2018-211881.

5. Schroeder JD, McKenzie AS, Zach JA, et al. Relationships Between Airflow Obstruction and Quantitative CT Measurements of Emphysema, Air Trapping, and Airways in Subjects With and Without Chronic Obstructive Pulmonary Disease. American Journal of Roentgenology 2013;201(3):W460–W470. DOI: 10.2214/AJR.12.10102.

6. Han MK, Kazerooni EA, Lynch DA, et al. Chronic Obstructive Pulmonary Disease Exacerbations in the COPDGene Study: Associated Radiologic Phenotypes. Radiology 2011;261(1):274–282. DOI: 10.1148/radiol.11110173.

7. Busacker A, Newell JD, Jr., Keefe T, et al. A multivariate analysis of risk factors for the air-trapping asthmatic phenotype as measured by quantitative CT analysis. Chest 2009;135(1):48–56. DOI: 10.1378/chest.08-0049.

8. Hersh CP, Hokanson JE, Lynch DA, et al. Family history is a risk factor for COPD. Chest 2011;140(2):343–350. DOI: 10.1378/chest.10-2761.

9. Galban CJ, Boes JL, Bule M, et al. Parametric response mapping as an indicator of bronchiolitis obliterans syndrome after hematopoietic stem cell transplantation. Biol Blood Marrow Transplant 2014;20(10):1592–8. DOI: 10.1016/j.bbmt.2014.06.014.

10. Labaki WW, Gu T, Murray S, et al. Voxel-Wise Longitudinal Parametric Response Mapping Analysis of Chest Computed Tomography in Smokers. Acad Radiol 2018. DOI: 10.1016/j.acra.2018.05.024.

11. Nakano Y, Wong JC, de Jong PA, et al. The prediction of small airway dimensions using computed tomography. Am J Respir Crit Care Med 2005;171(2):142–6. DOI: 10.1164/rccm.200407-874OC.

12. Miller MR, Hankinson J, Brusasco V, et al. Standardisation of spirometry. Eur Respir J 2005;26(2):319–38. (In eng). DOI: 10.1183/09031936.05.00034805.

13. Halpin DMG, Criner GJ, Papi A, et al. Global Initiative for the Diagnosis, Management, and Prevention of Chronic Obstructive Lung Disease. The 2020 GOLD Science Committee Report on COVID-19 and Chronic Obstructive Pulmonary Disease. Am J Respir Crit Care Med 2021;203(1):24–36. (In eng). DOI: 10.1164/rccm.202009-3533SO.

14. Gold L, Walker JJ, Wilcox SK, Williams S. Advances in human proteomics at high scale with the SOMAscan proteomics platform. N Biotechnol 2012;29(5):543–9. (In eng). DOI: 10.1016/j.nbt.2011.11.016.

15. Candia J, Cheung F, Kotliarov Y, et al. Assessment of Variability in the SOMAscan Assay. Scientific reports 2017;7(1):14248. (In eng). DOI: 10.1038/s41598-017-14755-5.

16. Candia J, Daya GN, Tanaka T, Ferrucci L, Walker KA. Assessment of variability in the plasma 7k SomaScan proteomics assay. Scientific reports 2022;12(1):17147. (In eng). DOI: 10.1038/s41598-022-22116-0.

17. Arjomandi M, Zeng S, Chen J, et al. Changes in Lung Volumes with Spirometric Disease Progression in COPD. Chronic Obstr Pulm Dis 2023;10(3):270–285. (In eng). DOI: 10.15326/jcopdf.2022.0363.

18. McKleroy W, Shing T, Anderson WH, et al. Longitudinal Follow-Up of Participants With Tobacco Exposure and Preserved Spirometry. JAMA 2023;330(5):442–453. DOI: 10.1001/jama.2023.11676.

19. Maloney J, Narasimhan J, Biller J. Decreased TGF-β1 and VEGF Release in Cystic Fibrosis Platelets: Further Evidence for Platelet Defects in Cystic Fibrosis. Lung 2016;194. DOI: 10.1007/s00408-016-9925-9.

20. Oyama Y, Shuff S, Davizon-Castillo P, Clendenen N, Eckle T. Intense light as anticoagulant therapy in humans. PLOS ONE 2020;15:e0244792. DOI: 10.1371/journal.pone.0244792.

21. Arjomandi M, Zeng S, Barjaktarevic I, et al. Radiographic lung volumes predict progression to COPD in smokers with preserved spirometry in SPIROMICS. Eur Respir J 2019;54(4) (In eng). DOI: 10.1183/13993003.02214-2018.

22. Arjomandi M, Zeng S, Barjaktarevic I, et al. Phenotypes and Trajectories of Tobacco-exposed Persons with Preserved Spirometry: Insights from Lung Volumes. Ann Am Thorac Soc 2024 (In eng). DOI: 10.1513/AnnalsATS.202405-527OC.

23. Zeng S, Tham A, Bos B, Jin J, Giang B, Arjomandi M. Lung volume indices predict morbidity in smokers with preserved spirometry. Thorax 2018. DOI: 10.1136/thoraxjnl-2018-211881.

24. Välikangas T, Suomi T, Elo LL. A systematic evaluation of normalization methods in quantitative label-free proteomics. Brief Bioinform 2018;19(1):1–11. (In eng). DOI: 10.1093/bib/bbw095.

25. Huber W, von Heydebreck A, Sültmann H, Poustka A, Vingron M. Variance stabilization applied to microarray data calibration and to the quantification of differential expression. Bioinformatics 2002;18 Suppl 1:S96–104. (In eng). DOI: 10.1093/bioinformatics/18.suppl_1.s96.

26. Bates D, Mächler M, Bolker B, Walker S. Fitting Linear Mixed-Effects Models Using lme4. Journal of Statistical Software 2015;67(1):1 – 48. DOI: 10.18637/jss.v067.i01.

27. Ulgen E, Ozisik O, Sezerman OU. pathfindR: An R Package for Comprehensive Identification of Enriched Pathways in Omics Data Through Active Subnetworks. Front Genet 2019;10:858. (In eng). DOI: 10.3389/fgene.2019.00858.

28. Gu Z, Eils R, Schlesner M. Complex heatmaps reveal patterns and correlations in multidimensional genomic data. Bioinformatics 2016;32(18):2847–2849. DOI: 10.1093/bioinformatics/btw313.

29. Stone M. An Asymptotic Equivalence of Choice of Model by Cross-Validation and Akaike's Criterion. Journal of the Royal Statistical Society: Series B (Methodological) 1977;39(1):44–47. DOI: https://doi.org/10.1111/j.2517-6161.1977.tb01603.x.

30. Lewis MJ, Spiliopoulou A, Goldmann K, Pitzalis C, McKeigue P, Barnes MR. nestedcv: an R package for fast implementation of nested cross-validation with embedded feature selection designed for transcriptomics and high-dimensional data. Bioinform Adv 2023;3(1):vbad048. (In eng). DOI: 10.1093/bioadv/vbad048.

31. Kuhn M. Building Predictive Models in R Using the caret Package. Journal of Statistical Software 2008;28(5):1 – 26. DOI: 10.18637/jss.v028.i05.

32. Chen T, Guestrin C. XGBoost: A Scalable Tree Boosting System. Proceedings of the 22nd ACM SIGKDD International Conference on Knowledge Discovery and Data Mining. San Francisco, California, USA: Association for Computing Machinery; 2016:785–794.

33. Breiman L. Random Forests. Machine Learning 2001;45(1):5–32. DOI: 10.1023/A:1010933404324.

34. Weihs C, Ligges U, Luebke K, Raabe N. klaR Analyzing German Business Cycles. In: Baier D, Decker R, Schmidt-Thieme L, eds. Data Analysis and Decision Support. Berlin, Heidelberg: Springer Berlin Heidelberg; 2005:335–343.
