# Supplementary figures and images for: Plasma proteomic profiles of lung volume–based phenotypes in tobacco-exposed individuals without spirometric chronic obstructive pulmonary disease
Source: Ann Am Thorac Soc. 2026 Mar 3;23(7):1033–45. doi: 10.1093/annalsats/aaoag051 (PMC13315777; doi:10.1093/annalsats/aaoag051)

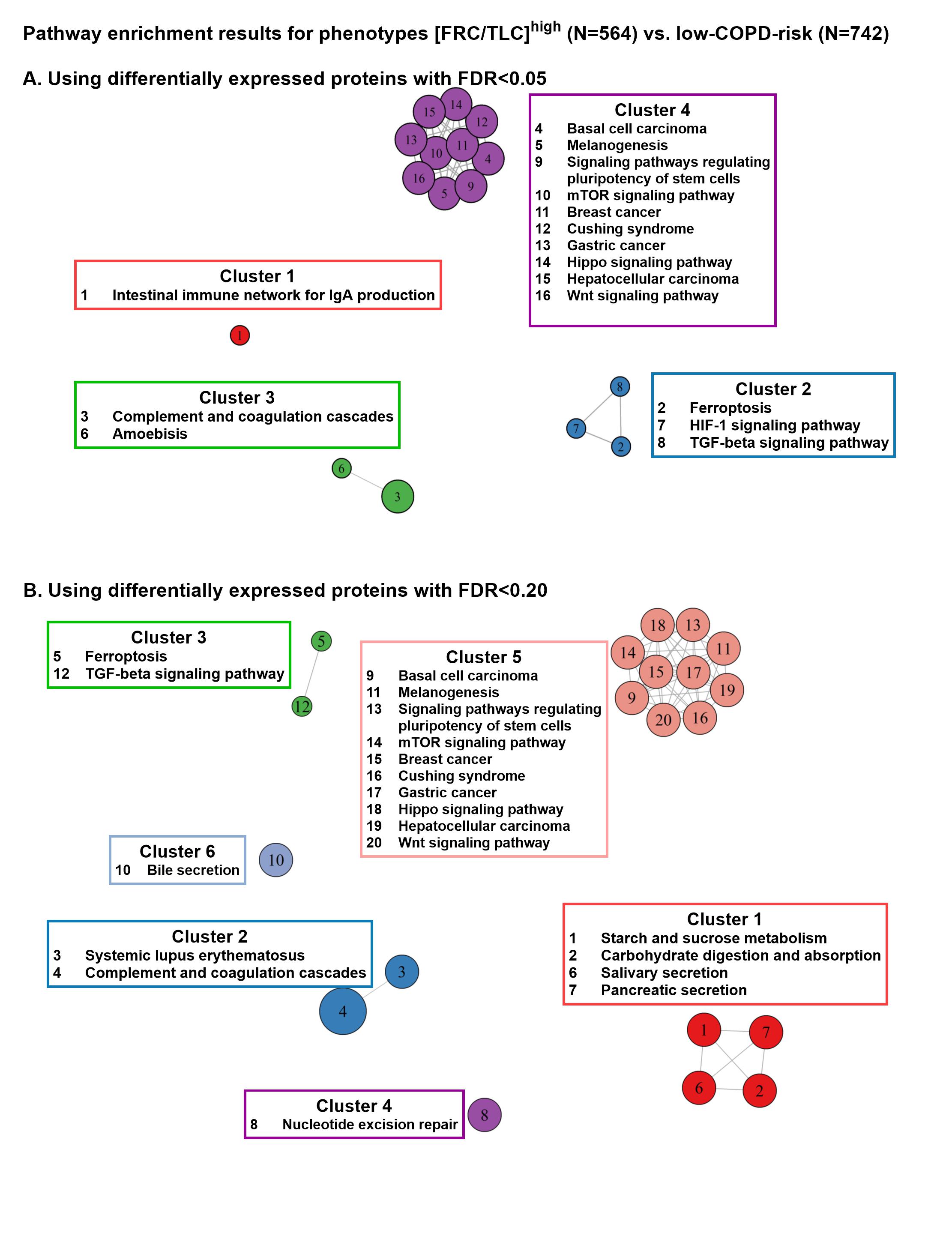

Supplement: aaoag051_Supplementary_Data [file aaoag051_supplementary_data.zip › Fiugre S13-Pathway network FRCTLC0.2.vs.jpg]

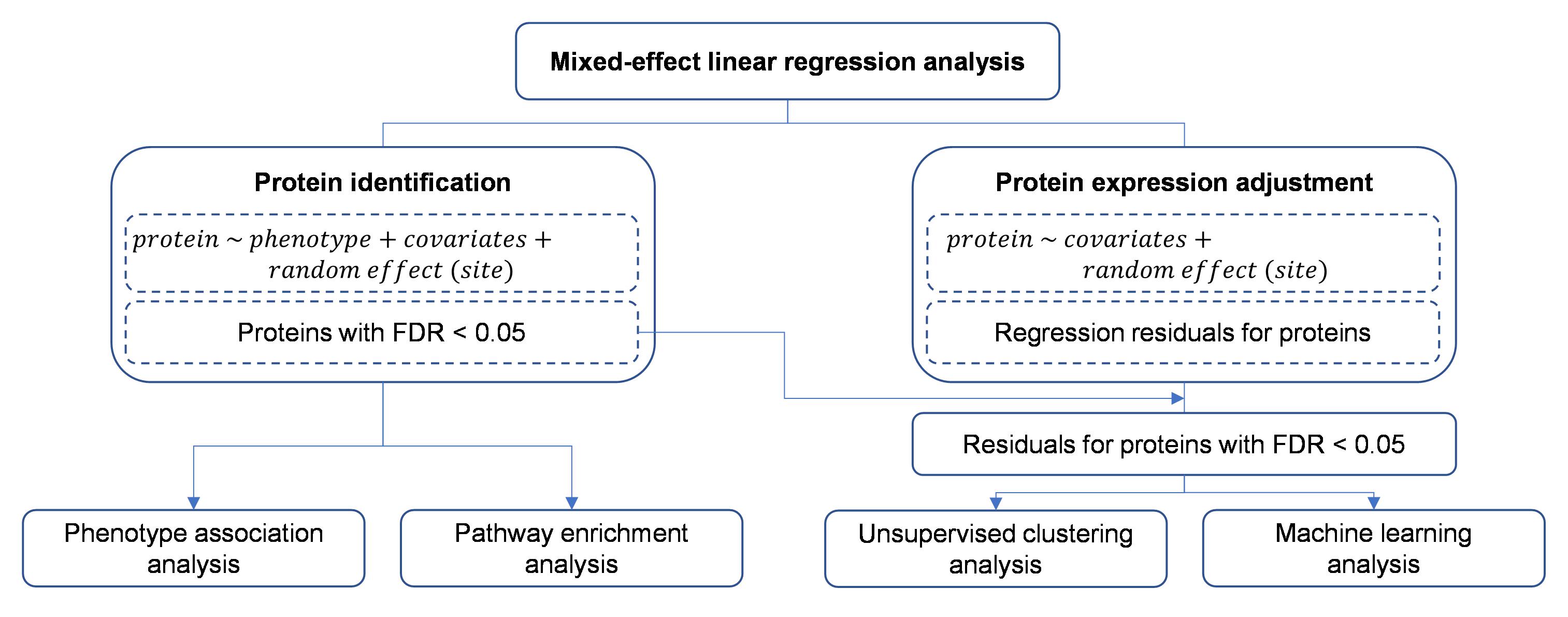

Supplement: aaoag051_Supplementary_Data [file aaoag051_supplementary_data.zip › Figure S1-Analysis flow.jpg]

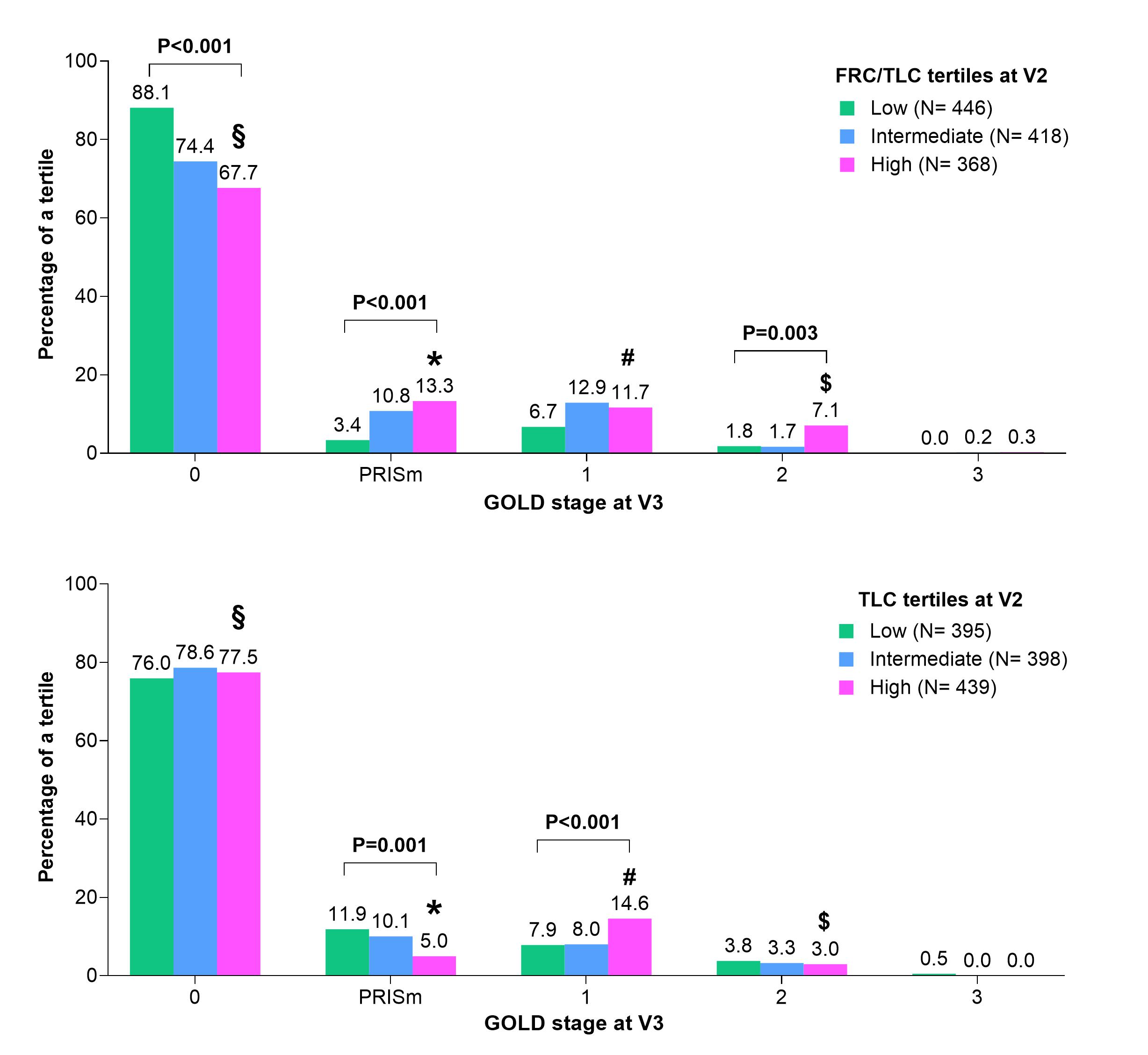

Supplement: aaoag051_Supplementary_Data [file aaoag051_supplementary_data.zip › Figure S2-Follow-up counts.jpg]

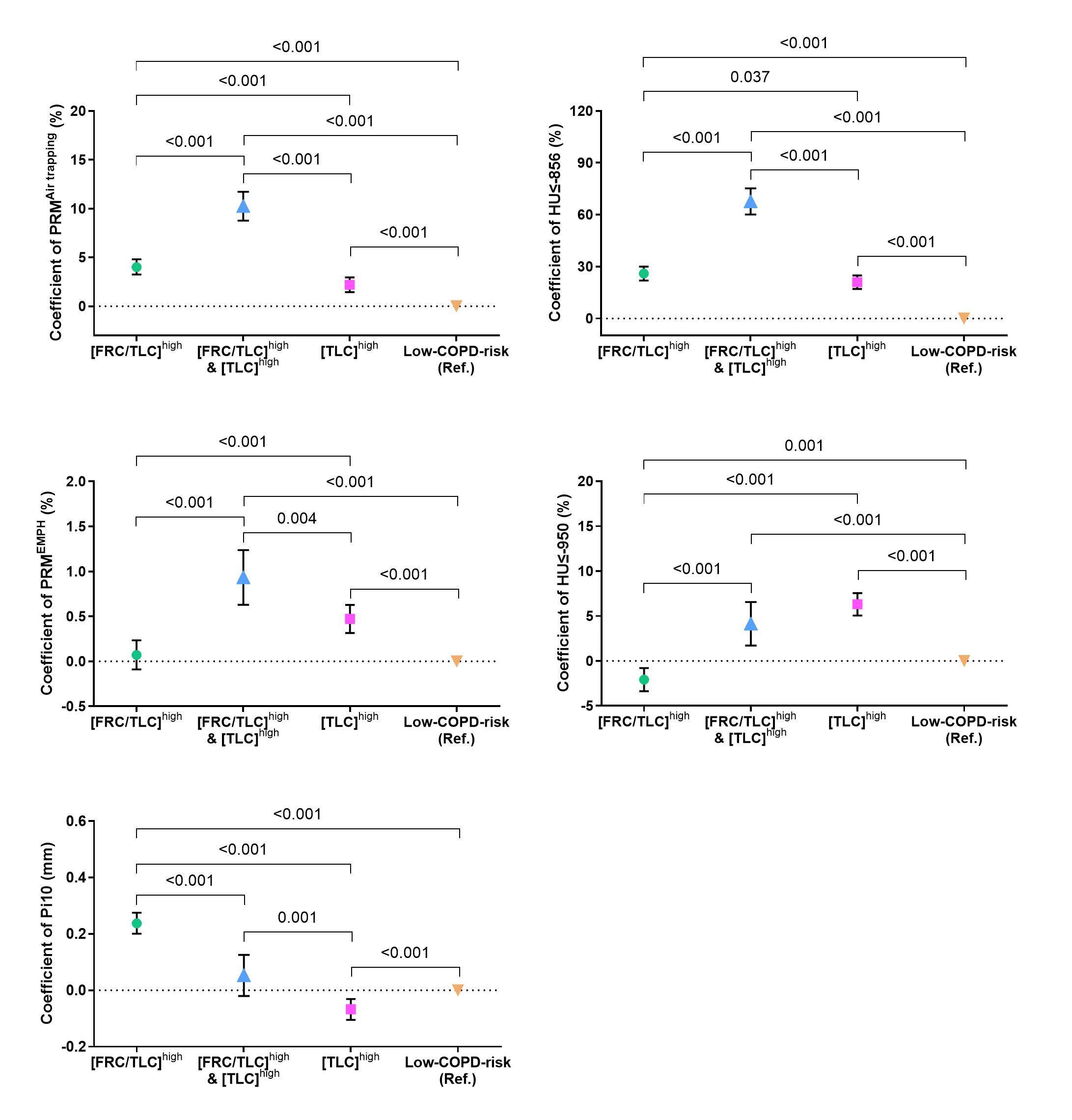

Supplement: aaoag051_Supplementary_Data [file aaoag051_supplementary_data.zip › Figure S3-CT 4G.jpg]

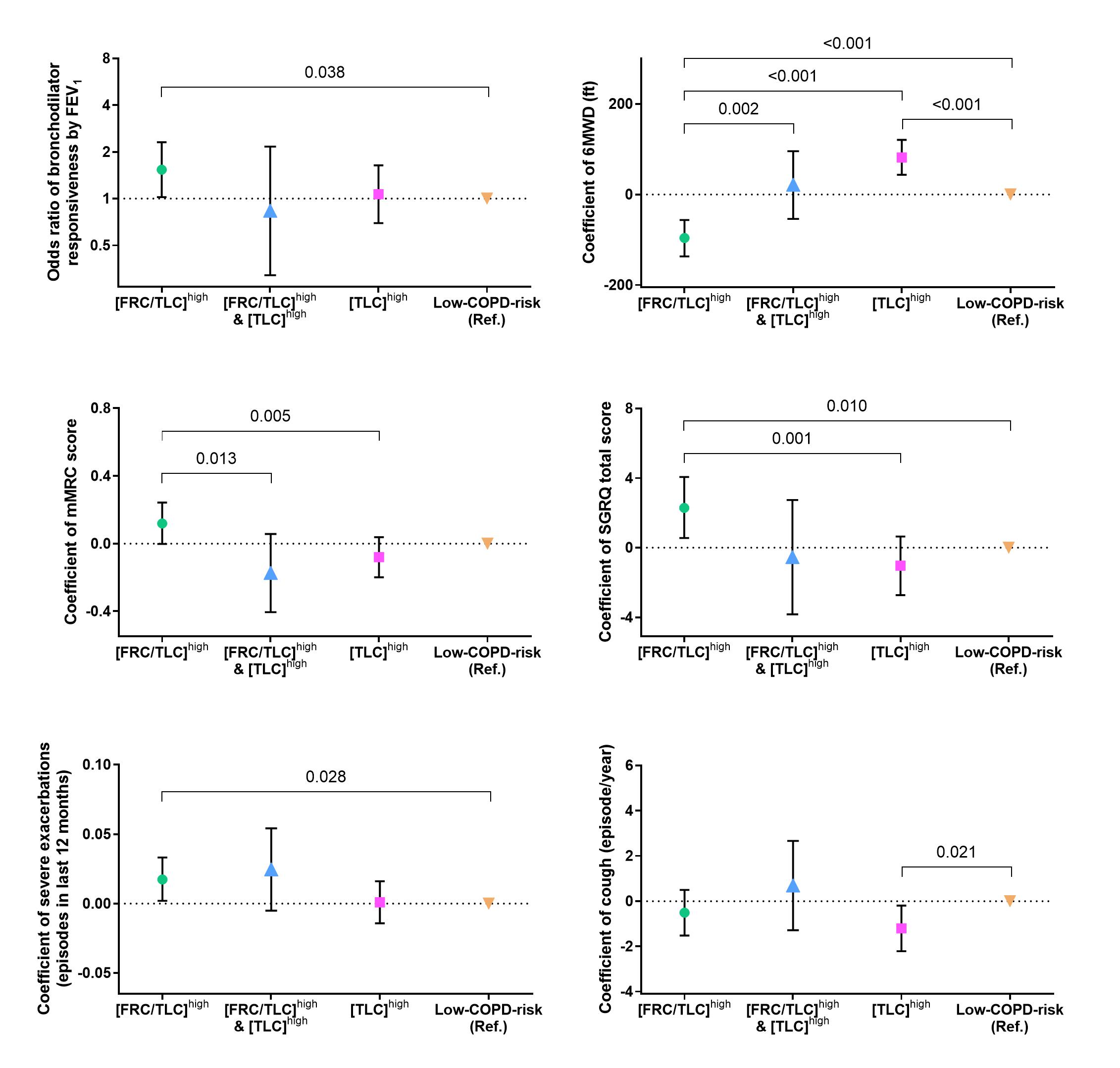

Supplement: aaoag051_Supplementary_Data [file aaoag051_supplementary_data.zip › Figure S4-Clinical 4G.jpg]

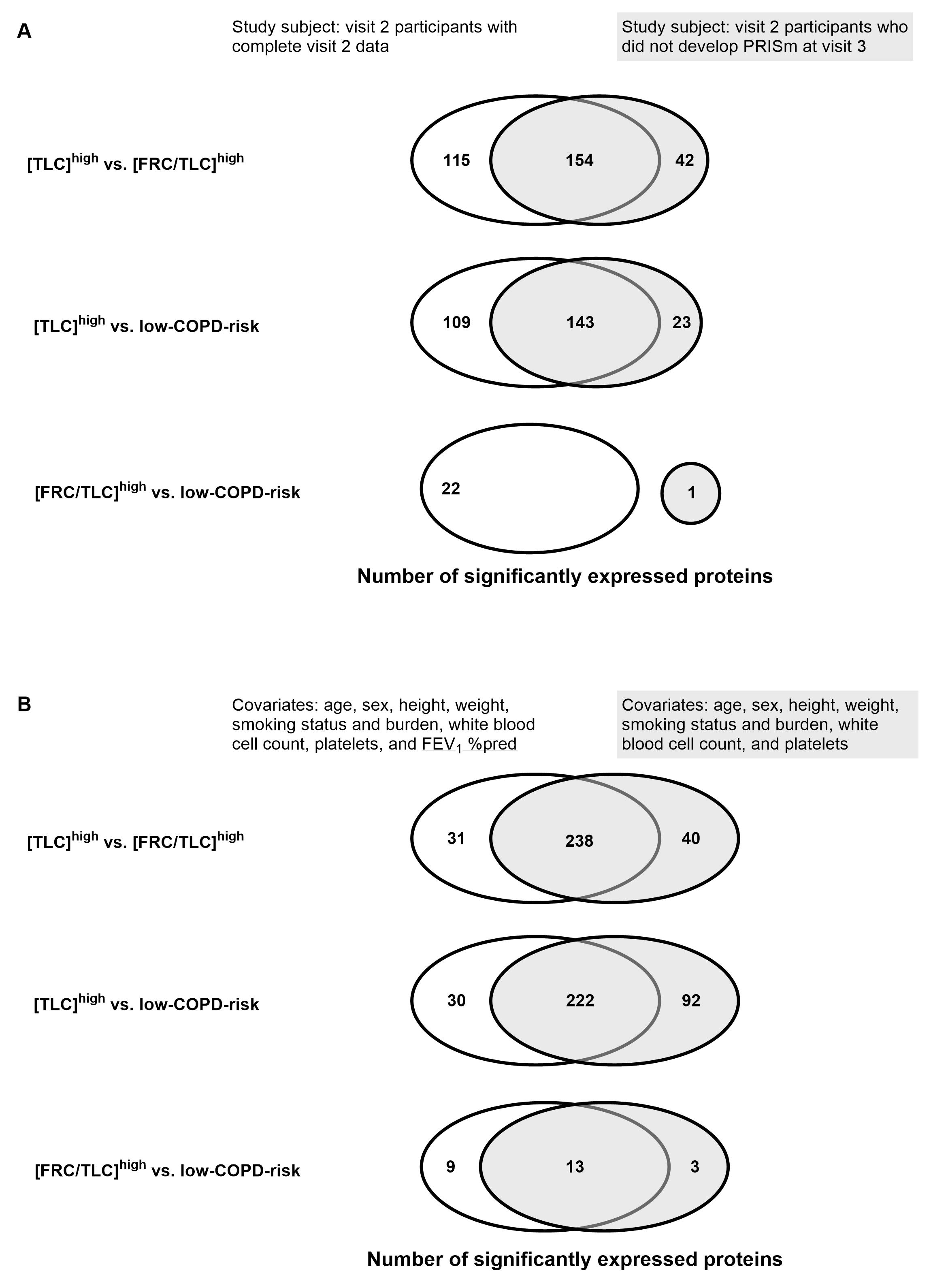

Supplement: aaoag051_Supplementary_Data [file aaoag051_supplementary_data.zip › Figure S5-Sensitivity_n_Proteins.jpg]

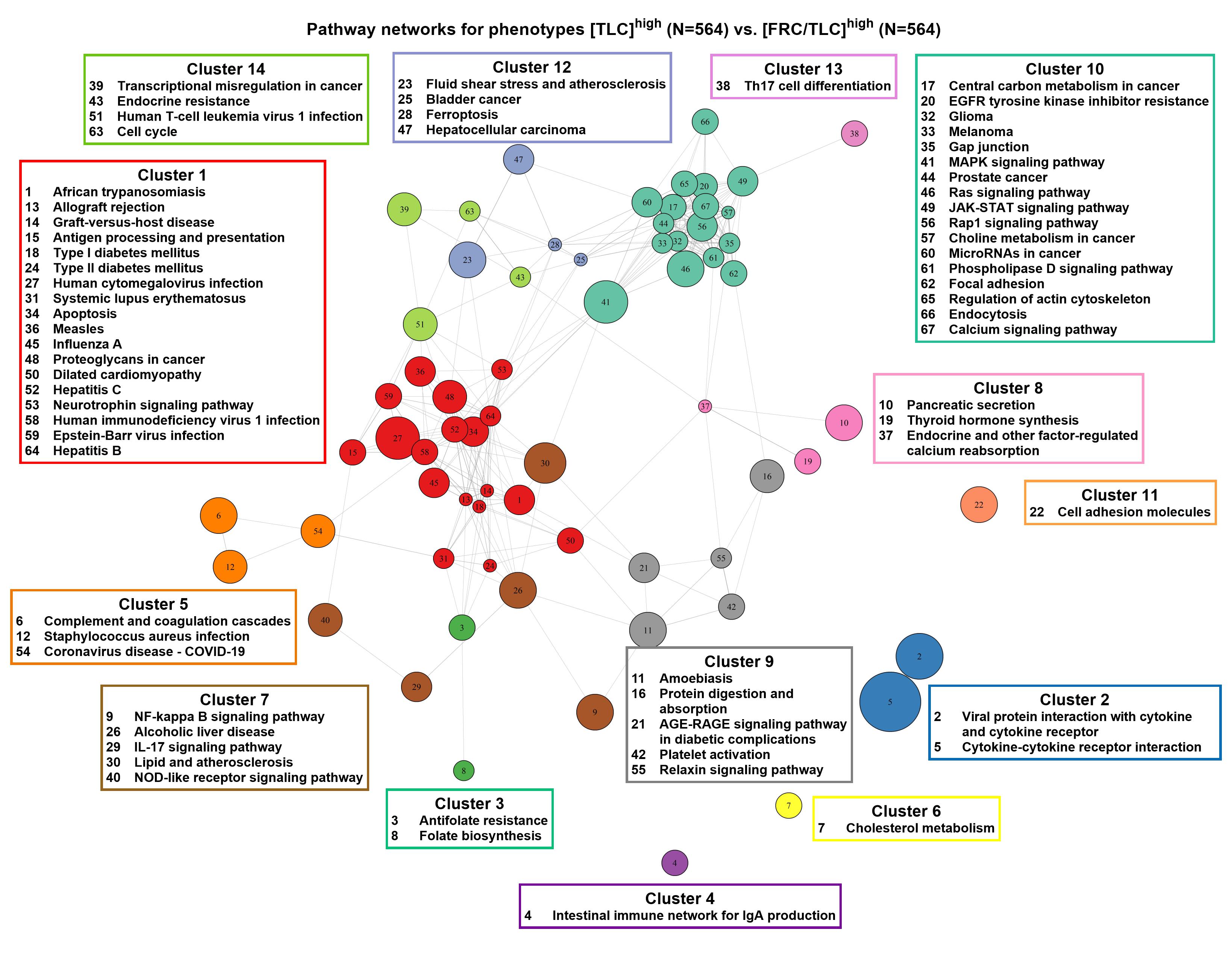

Supplement: aaoag051_Supplementary_Data [file aaoag051_supplementary_data.zip › Figure S6-Pathway network.jpg]

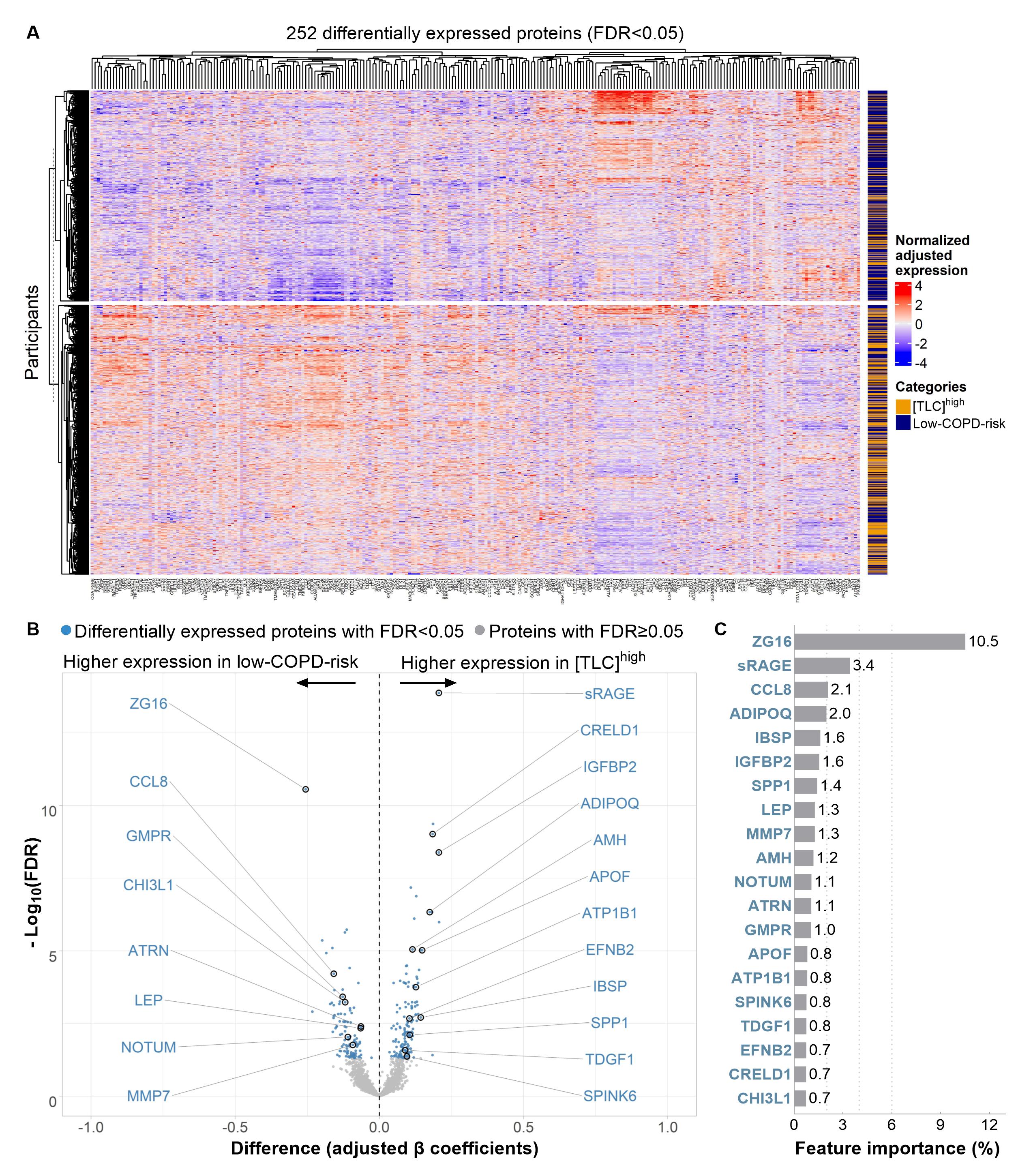

Supplement: aaoag051_Supplementary_Data [file aaoag051_supplementary_data.zip › Figure S7-Markers TLC.vs.jpg]

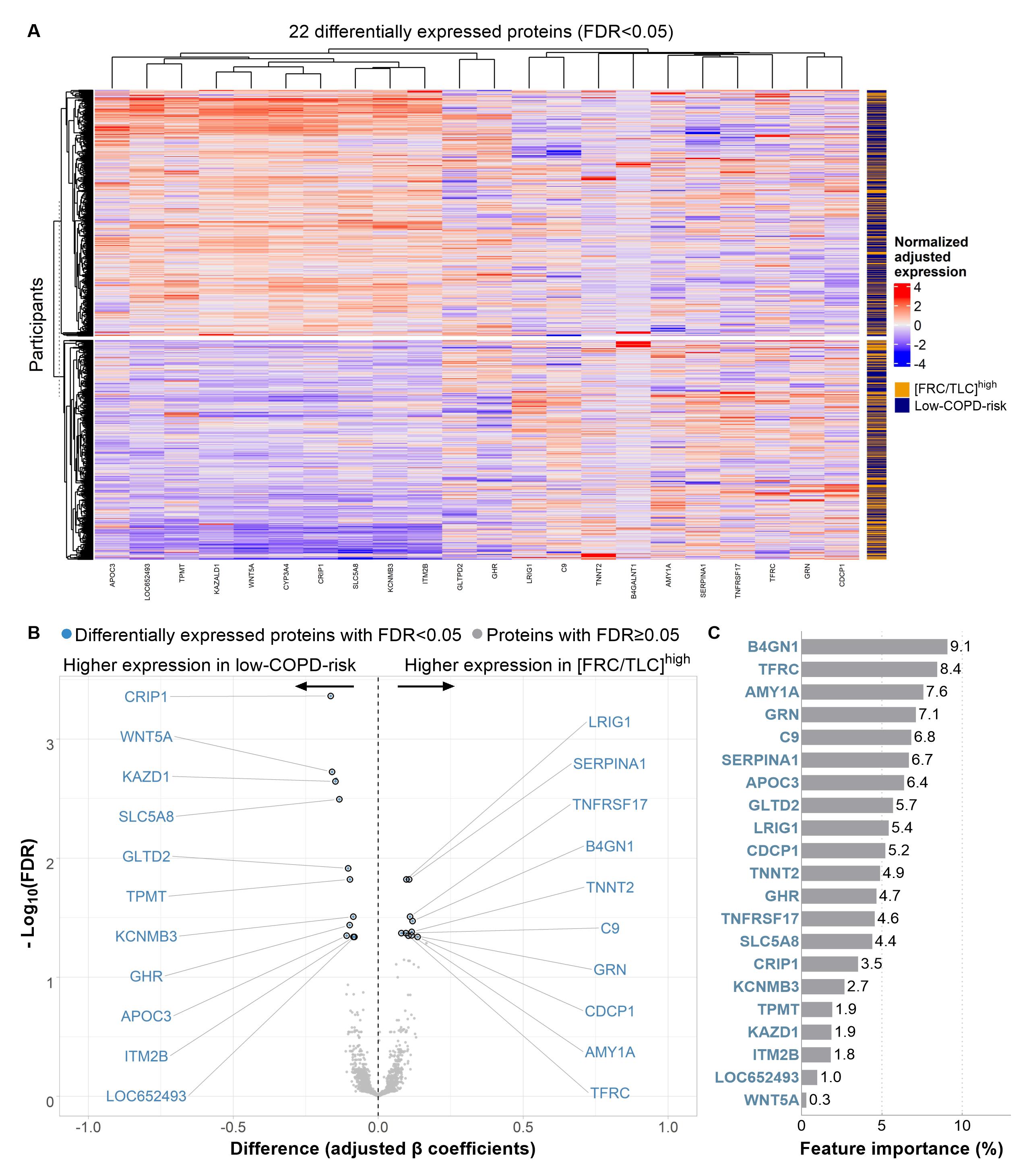

Supplement: aaoag051_Supplementary_Data [file aaoag051_supplementary_data.zip › Figure S8-Markers FRCTLC.vs.jpg]

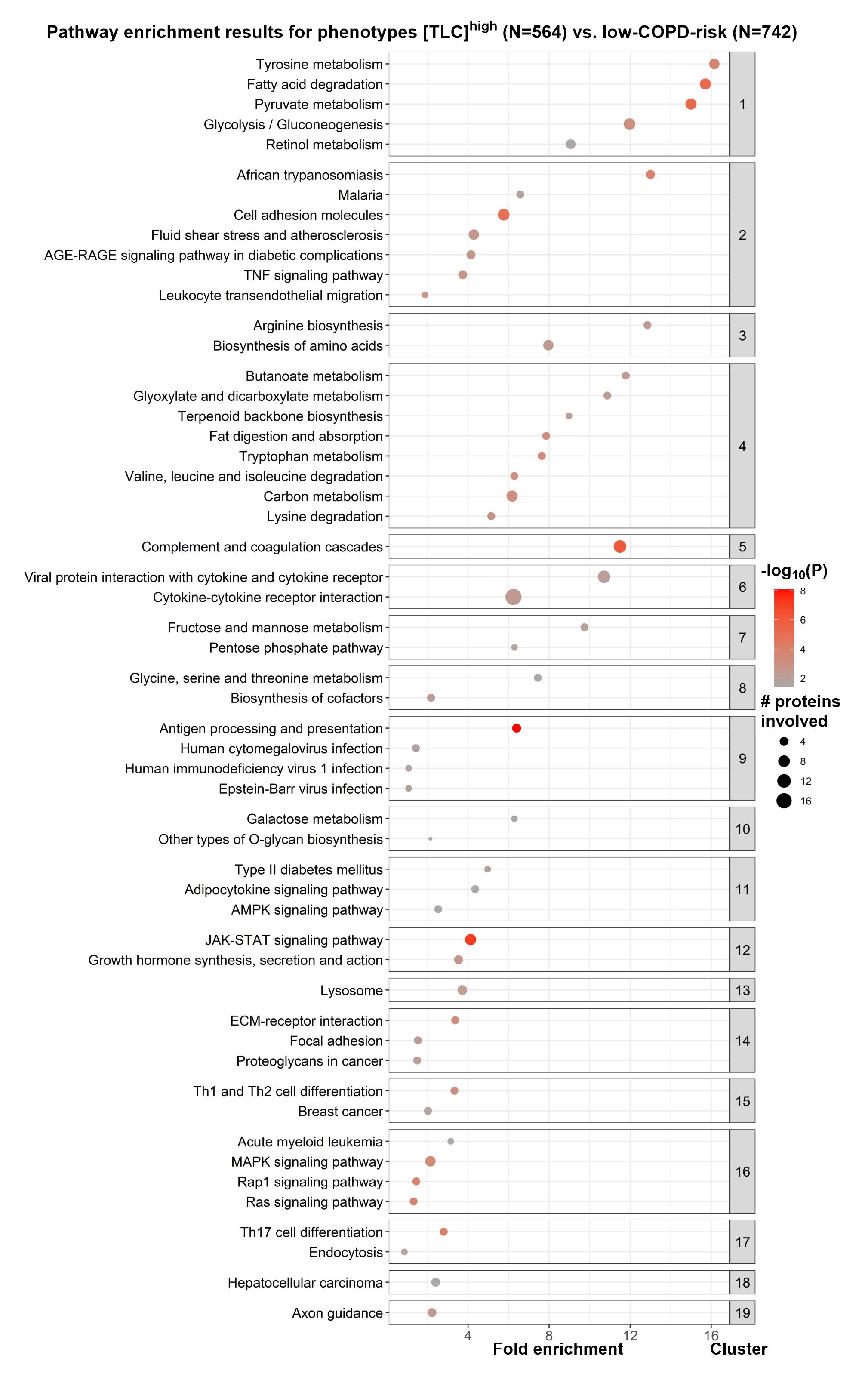

Supplement: aaoag051_Supplementary_Data [file aaoag051_supplementary_data.zip › Figure S9-Pathway chart TLC.vs.jpg]

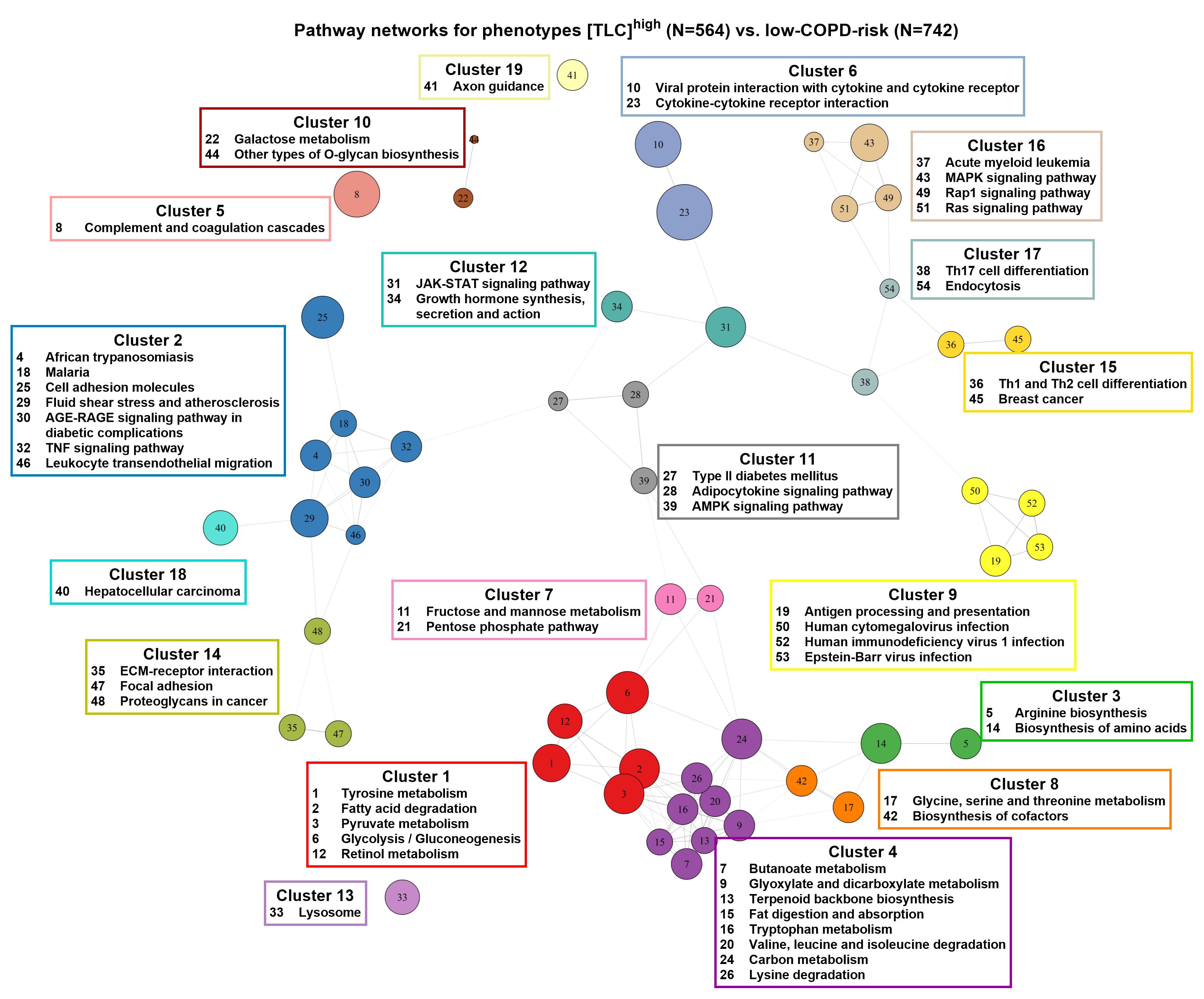

Supplement: aaoag051_Supplementary_Data [file aaoag051_supplementary_data.zip › Figure S10-Pathway network TLC.vs.jpg]

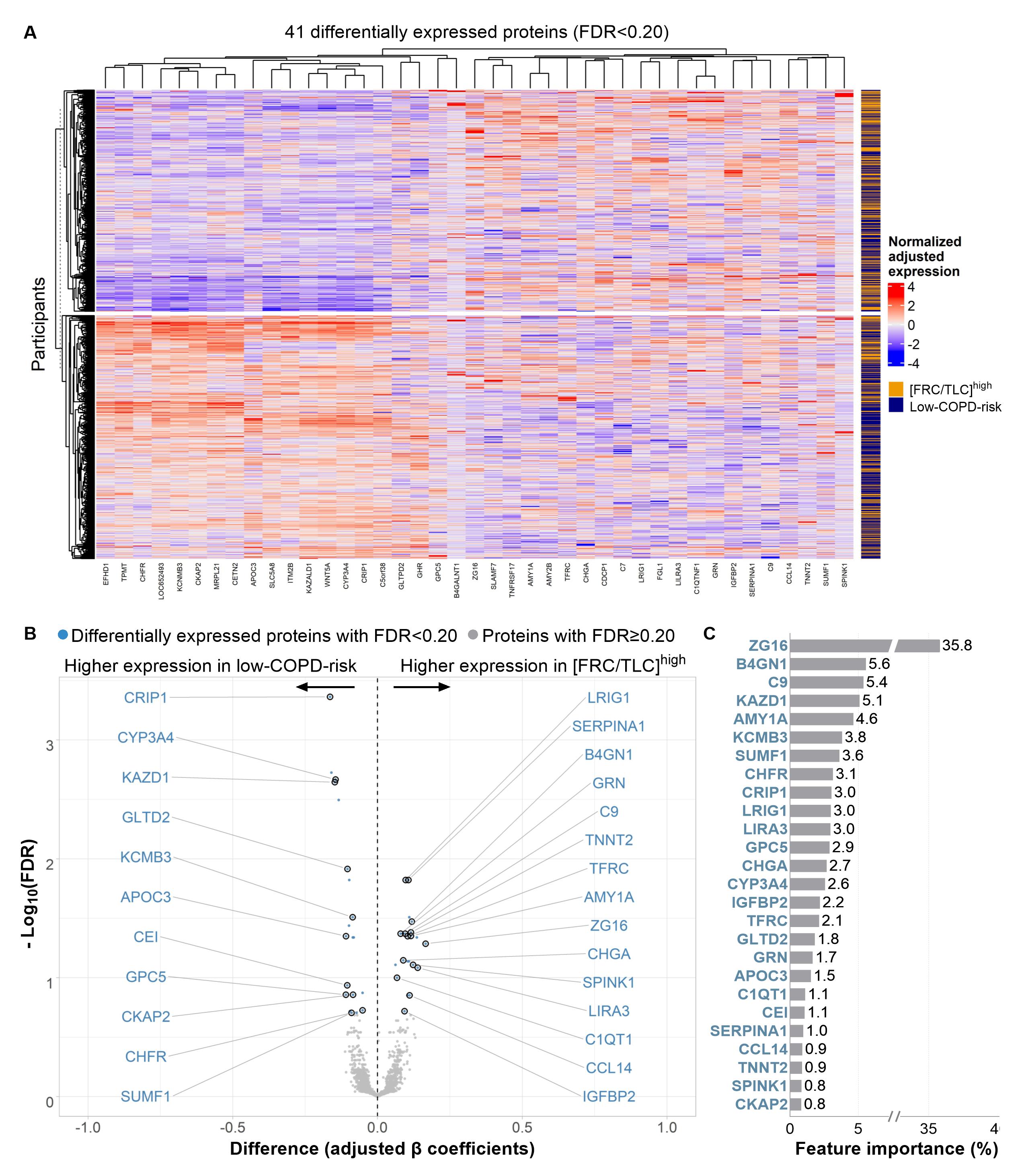

Supplement: aaoag051_Supplementary_Data [file aaoag051_supplementary_data.zip › Figure S11-Markers FRCTLC0.2.vs.jpg]

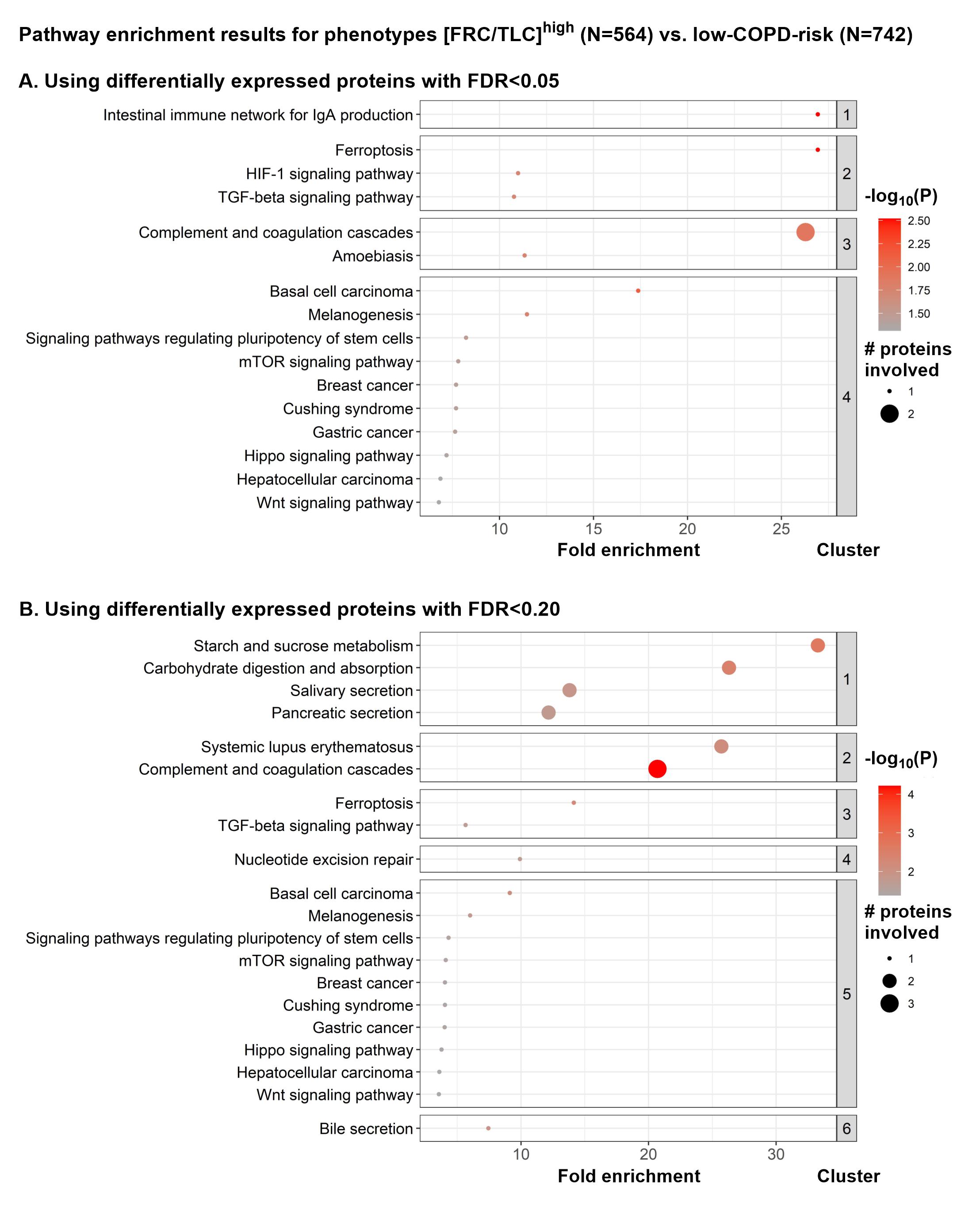

Supplement: aaoag051_Supplementary_Data [file aaoag051_supplementary_data.zip › Figure S12-Pathway chart FRCTLC0.2.vs.jpg]
